# The inhibitory effect of intraspinal microstimulation of the sacral spinal cord on nonlinear bladder reflex dynamics in cats

**DOI:** 10.3389/fnins.2025.1519377

**Published:** 2025-02-03

**Authors:** Amirhossein Qasemi, Alireza Aminian, Abbas Erfanian

**Affiliations:** Department of Biomedical Engineering, School of Electrical Engineering, Iran Neural Technology Research Center, Iran University of Science and Technology, Tehran, Iran

**Keywords:** intraspinal microstimulation, incontinence, bladder inhibition, neuromodulation, cat, chaotic dynamics, recurrence plot

## Abstract

**Objective:**

Electrical stimulation of the pudendal nerve, pelvic nerve, sacral dorsal root ganglion (DRG), and spinal cord has been explored to treat urinary incontinence and overactive bladder (OAB). This study introduces sacral intraspinal microstimulation (ISMS) as a novel method to inhibit spontaneous bladder reflexes in anesthetized cats. In addition, we investigated the effects of intermittent and switching stimulation patterns on bladder inhibition.

**Methods:**

The electrode was implanted in the dorsal horn of the S2 spinal cord. Bladder pressure was recorded under isovolumetric conditions, and the stimulation parameters were adjusted to inhibit spontaneous bladder contractions. Nonlinear dynamic methods, including chaos theory, were employed to analyze the complexity of bladder reflexes.

**Results:**

Results demonstrated that ISMS targeting the dorsal horn of the S2 spinal segment effectively suppressed high-amplitude spontaneous contractions. Furthermore, bladder reflexes exhibited complex dynamics, ranging from regular to chaotic patterns, with transitions between these states. Importantly, ISMS was able to stabilize these chaotic dynamics, leading to more controlled bladder behavior.

**Conclusion:**

These findings suggest that sacral ISMS offers a promising, targeted alternative to traditional stimulation therapies, potentially providing a new therapeutic approach for managing OAB and urinary incontinence by regulating chaotic bladder activity.

## 1 Introduction

The lower urinary tract (LUT), which consists of the bladder, urethra, and external urethral sphincter (EUS), has two main functions: storing and periodically eliminating urine. These functions are controlled by neural circuits in the brain and the spinal cord (Beckel and Holstege, 2011; de Groat et al., 2014). Various disorders impair normal LUT function and can be divided into two categories: voiding disorders, such as detrusor sphincter dyssynergia (DSD) and underactive bladder, and storage disorders, such as urinary urgency incontinence (UUI) and overactive bladder (OAB) (Coolen et al., 2019; de Groat et al., 2014; McGee et al., 2015). According to the International Continence Society (ICS) definition, OAB is “urgency, with or without urge incontinence, usually with frequency and nocturia in the absence of proven infection or other obvious pathology” (Abrams et al., 2003).

The general approach to treating incontinence is to rule out reversible causes of urinary incontinence first and then continue with conservative management and pharmacological management, followed by specialized management (Liao and Madersbacher, 2019). When these treatment methods do not provide adequate health benefits, electrical stimulation has been proposed as an effective approach for enhancing bladder functions in patients with neurogenic LUT dysfunction (Coolen et al., 2019; de Groat et al., 2014; Liao and Madersbacher, 2019). Sacral neuromodulation (SNM) and posterior tibial nerve stimulation (PTNS) are two US Food and Drug Administration (FDA)-approved treatments for OAB (Coolen et al., 2019; Liao and Madersbacher, 2019). In SNM, electrical stimulation is delivered through a lead placed in the third or fourth sacral foramen to target somatic fibers entering the spinal cord. In one study, after the 1-month follow-up, 87% of women with refractory idiopathic urgency urinary incontinence experienced a 50% or greater reduction in the number of incontinence episodes or daily pad use. This number decreased to 62% at 5 years. In 15% of the patients, complete continence was achieved throughout the follow-up period. However, SNM is mostly used in nonneurogenic patients. The PTNS procedure is less invasive than SNM and is performed with an electrode inserted above the ankle (Coolen et al., 2019).

Electrical stimulation of the pudendal nerve and its branches is an alternative approach to restoring bladder function in rats and cats (Boggs et al., 2006b; Hokanson et al., 2021). The pudendal nerve, which is composed of sensory (SN) and motor fibers, arises from spinal nerves S2-S4, which innervate the pelvic floor muscles, the external urethral and anal sphincters, and the pelvic organs (Spinelli et al., 2005). The SN continues to branch off into the cranial urethral sensory nerve (CSN) and the dorsal nerve of the penis (DNP), whereas the RP splits into the deep perineal (DPN) and caudal rectal (CR) nerves (Yoo et al., 2008a; Yoo et al., 2008b).

Electrical stimulation of the compound pudendal nerve can evoke, depending on the stimulation frequency, either an excitatory effect through afferent fiber activation or an inhibitory effect through modulation of the efferent fibers (Boggs et al., 2006b; Tai et al., 2006; Tai et al., 2007a; Tai et al., 2011). Low-frequency (10 Hz) stimulation can inhibit bladder contraction and activate the EUS, but mid-frequency (33 Hz) stimulation can excite the bladder without activation of the EUS (Boggs et al., 2006b). Additionally, the inhibitory effect is maximal at a stimulation frequency of 3 Hz (Tai et al., 2006; Tai et al., 2007a) and gradually decreases at lower or higher frequencies (Tai et al., 2006). In cats with chronic SCI, stimulation at 3–10 Hz inhibits bladder isovolumetric rhythmic contractions and increases bladder capacity to 180% of control capacity in cystometrogram (CMG) trials (Tai et al., 2011).

In one study (Shapiro et al., 2020), the effect of bilateral stimulation of the pudendal nerve on cats was investigated, and it was reported that stimulation at 5 Hz and 2 times the threshold intensity increased the bladder capacity by 60%, whereas unilateral stimulation resulted in only a 30% increase. In contrast, stimulation with the threshold intensity did not significantly enhance bladder capacity.

To maximize the therapeutic efficacy of pudendal nerve stimulation on bladder capacity, the effect of direct stimulation of the pudendal sensory branch was also investigated (Hokanson et al., 2018; Hokanson et al., 2021). Sensory branch stimulation significantly increased bladder capacity in urethane anesthetized female rats; however, it decreased the efficiency of subsequent voiding due to strong bladder inhibition (Hokanson et al., 2018). To overcome the stimulation carryover effect, pudendal sensory branch stimulation (10 Hz) was followed by pudendal motor branch bursting stimulation (Hokanson et al., 2021). This combined stimulation could lead to a significant increase in bladder capacity and enhance the voiding efficiency. However, the feasibility of implanting two cuff electrodes on two tiny nerves (especially sensory nerves) is challenging.

High-frequency (kHz) stimulation (HFS) of the sacral roots or pudendal nerve could prevent EUS activation and allow bladder voiding in patients with DSD (Bhadra et al., 2006; Boger et al., 2007; Boger et al., 2012; Tai et al., 2004b; Tai et al., 2005; Tai et al., 2007b). Unilateral HFS of the pelvic nerve can inhibit bladder voiding (Crook and Lovick, 2017). High-frequency (1–3 kHz) sinusoidal pelvic nerve stimulation is triggered at the onset of increased bladder pressure, suppressed urine output and increased EUS tone. However, HFS has a nonphysiological pattern in which a high amount of charge is delivered to the tissue, which may cause nerve damage (Tai et al., 2004b). Although the amount of charge injected during HFS is within the range of the safe limit (Bhadra et al., 2006), it is not clear that HFS would be within the range of the safety limit that was determined according to the criteria established for frequencies below 50 Hz in research (Merrill et al., 2005). Moreover, this approach may cause faster electrode oxidation and high consumption of power, which is a concern in implantable devices.

Pelvic nerve stimulation (PelN) has been shown to be effective in increasing the bladder capacity of OAB subjects (Langdale et al., 2017; Langdale et al., 2020) in both rats and cats. Prostaglandin E2 (PGE2) infusion has been used as a model of OAB that results in decreased bladder capacity (BC). PelN stimulation reversed PGE2-induced reductions in BC; however, the stimulation frequency differed between rats (10 Hz) and cats (1 Hz). Although peripheral nerve stimulation is promising, it is very prone to lead migration (Liem, 2015; Wang et al., 2018a), surgical access to the target nerve is sometimes difficult, and it suffers from unrelated efferent fiber activation.

Sacral dorsal root ganglion (DRG) stimulation at a low frequency (3–7 Hz, S1 or S2) significantly inhibited isovolumetric rhythmic bladder contractions, whereas stimulation of the sacral DRG at a lower frequency (0.25–1.5 Hz) and middle frequency (15–30 Hz) evoked bladder contraction (Wang et al., 2018a). Further research revealed that S1 and S2 DRG stimulation can increase bladder capacity in both intact and OAB (Wang et al., 2018b). Although the sacral DRG is located near the spinal cord column and is more immune with respect to mechanical force than the pudendal nerve trunk is (Liem, 2015; Wang et al., 2018a), the suspended structure of the dorsal root ganglia and the attachment of an array to it increase the risk of tearing the roots. Moreover, the sacral DRG is located near the pelvis in the caudal space of the spinal cord, which requires a much more invasive surgical approach for electrode implantation and may damage the subject’s skeleton (especially the sacrum) and impair normal movement function.

Spinal cord epidural stimulation (ScEs) has recently shown promising results in restoring bladder function in human (Herrity et al., 2018; Herrity et al., 2021; Walter et al., 2018) and animal (Hoey et al., 2021; Hoey et al., 2022; Jantz et al., 2022; Sysoev et al., 2020) subjects. ScEs, depending on electrode configuration and stimulation parameters (i.e., amplitude, frequency, and pulse width), can modulate detrusor pressure and external anal sphincter/pelvic floor muscle tone to various degrees during filling and voiding phase investigations (Walter et al., 2018). It was shown in a complete motor spinal cord injury subject that ScEs can improve voiding function (Herrity et al., 2018). In chronic rats, stimulation of the upper lumbar (L1) and lower lumbar (L5–L6) regions could activate mainly the detrusor muscle, and stimulation of the sacral region could activate predominantly the EUS (Sysoev et al., 2020).

Research on anesthetized intact rats has shown that high-frequency lumbosacral ScEs (L5–S1) with visualized movement threshold intensities in the filling phase could increase the inter contraction interval (ICI) and EUS tonic activity without an explosion of fluid but a short-latency void in chronically T9-transfected rats (Hoey et al., 2021). These results were similar to the effects of thoracolumbar epidural stimulation, but the detrusor pressure remained low during longer ICI and did not increase as it did during L5–S1 scES (Hoey et al., 2022). This theory that sacral ScEs with a high-density epidural electrode array may be utilized to specifically recruit LUT nerves was tested in another investigation on isoflurane-anesthetized cats (Jantz et al., 2022). Sacral epidural stimulation and concurrent nerve activity monitoring in cats demonstrated that sacral ScEs elicit reactions in the nerves innervating the bladder and urethra and that these nerves can be selectively activated (Jantz et al., 2022). However, epidural stimulation has inherent limitations in achieving electrical field specificity (stimulation selectivity), resulting in stimulation spreading to unrelated muscle groups (Hachmann et al., 2021). This widespread dispersion of current can inadvertently activate various fibers linked to movement, as well as functions such as the bowel. Moreover, it is prone to lead migration when gravitational or mechanical forces caused by body movements alter the distance between the electrodes and the dorsal columns, leading to shifts in the perception of stimulation (Liem, 2015).

Many studies have used intraspinal microstimulation (ISMS) to restore bladder voiding function in intact and SCI cats (Carter et al., 1995; Grill et al., 1999; McCreery et al., 2004; Pikov et al., 2007; Pikov et al., 2020; Tai et al., 2004a) or intact rats (Yousefpour and Erfanian, 2021). Sacral parasympathetic nucleus (SPN) and dorsal gray commissure (DGC) stimulation at 20 Hz induce large bladder contractions and decrease urethral pressure, respectively, which mimics natural voiding (McCreery et al., 2004; Pikov et al., 2007; Pikov et al., 2020; Yousefpour and Erfanian, 2021). However, all the previous works regarding the use of the ISMS for restoring bladder function focused on voiding. For example (Pikov et al., 2007), in two intact cats, stimulation with a microelectrode located in the middle of the dorsal horn inhibited spontaneous bladder contractions. However, this effect was not quantified.

The major goal of this study is to quantify the effect of the sacral ISMS on bladder inhibition in intact alpha-chloralosed anesthetized cats under isovolumetric conditions. Despite the complex dynamic behavior (i.e., chaotic dynamics) of spontaneous bladder contraction, the ISMS could successfully suppress bladder contraction. We will demonstrate that the spinal bladder reflex exhibits complex dynamics (i.e., simple phasic contraction, complex phasic contraction, and complex tonic contraction). The ISMS could control the chaotic behavior of the spontaneous bladder reflex and stabilize the dynamics to an equilibrium point. Moreover, the effects of stimulation parameters (i.e., frequency and amplitude) and stimulation patterns (i.e., continuous stimulation, intermittent stimulation, and switching stimulation) on bladder inhibition during spontaneous bladder contractions during conditional stimulation were investigated and quantified.

## 2 Materials and methods

### 2.1 Experimental model and subject details

All surgical procedures and experimental protocols were approved by the Animal Care and Ethics Committee of Iran Neural Technology Research Centre, Iran University of Science and Technology. All protocols and methods were performed according to the recommendations and relevant guidelines for the care and use of laboratory animals. Moreover, the study was carried out in compliance with the ARRIVE guidelines.

Acute experiments were conducted on 27 male sexually intact adult domestic short-hair cats (10–22 months, 2.5–5.7 kg). Cats were chosen because of their physiological similarity to humans (especially LUT and the spinal cord) and the existence of numerous LUT studies on them (Fry et al., 2010). Animals with an irregular voiding period, feline herpesvirus, calicivirus, chlamydia, toxoplasmosis, and low weight compared with their body structure were excluded from the experiment before surgery. In one cat, the catheter was blocked, and fluid infusion into the bladder was interrupted. Two cats were also excluded because of a nonreflexive bladder after the initial dose of alpha-chloralose. In one cat, there was no response to the ISMS, and the reason was unclear. Finally, a feasibility study was performed with 9 cats to evaluate the critical aspects of the surgical procedure, anesthesia protocols, setup design (5 cats), and stimulation protocol (4 cats), followed by data collection from 6 cats under isovolumetric conditions. Moreover, contraction dynamics analysis utilized data from 8 cats. Prior to surgery, the animals were housed individually in a 12 m^2^ room with a moderate temperature (21–23°C) and a 12 h light-dark cycle. Food was provided twice a day, and the water and soil were changed daily and every other day, respectively. Animal urination and excretion were checked 72 h before surgery to ensure normal LUT function. Food (not water) was withheld for 12 h prior to the surgery. The experimental procedure in this study was terminal. At the end of the experiments, the animals were euthanized with potassium chloride (10 ml of 2 mEq/ml) under a high rate of isoflurane anesthesia.

### 2.2 Surgical preparation

Anesthesia was induced with isoflurane (initially with a chamber followed by a face mask, 5% in O_2_) and maintained during surgery with an endotracheal tube (1.5–2.5%). To facilitate the metabolism of isoflurane for terminal anesthesia, isoflurane anesthesia (anesthesia induction and surgery) was limited to a duration of less than three hours. Following surgery, anesthesia was gradually transitioned (over 40 min) from isoflurane to alpha-chloralose (Sigma C0128, Sigma-Aldrich; 60 mg/kg initial dose prepared in 1% solution at 90°C with heat for 30 min) at a rate of 0.4 ml/min to reduce the risk of acidosis for experimentation. A supplemental dose of alpha-chloralose (10 mg/kg) was injected during the experiment on the basis of blood pressure, eye reflex, and jaw tone to maintain anesthesia. Compared with other anesthetics, alpha-chloralose was chosen as the anesthetic because it minimally interferes with autonomic function and largely preserves spinal reflexes (Grill et al., 1999; Xu et al., 2020). Before surgery, gentamicin (5 mg/kg, IM) and ketoprofen (2 mg/kg, SQ) were injected.

The vital signs of the animals were monitored with a monitoring system (Alborz B5 system, Saadat Co., Iran). Airway access was secured with an endotracheal tube (Size 3, Biotek Medical Technology Co., China) connected to a pressure-regulated artificial respirator (LTV-950, Pulmonetic Systems, US) to maintain the end-tidal Co_2_ concentration between 3.8 and 4%, which was tracked via a mainstream gas analyzer (IRMA Co_2_ probe, Masimo, US). The right carotid artery was cannulated with a heparinized PE-90 tube (inner diameter 0.9 mm) connected to a pressure sensor (MX960, Smith Medical, UK) for continuous measurement of arterial blood pressure. Body temperature was measured via a probe (Saadat Co., Iran) mounted in the mouth and maintained at 37–38°C via a heating pad. Heart rate and SpO_2_ were measured by a pulse oximeter probe (2054, Masimo, US). The left cephalic vein was cannulated with a 22G angiocath (Supa, Iran) for intravenous injection and additional fluid administration (0.9% saline, 5% dextrose; 10 ml/kg/h).

### 2.3 Bladder catheter placement

**FIGURE 1:**
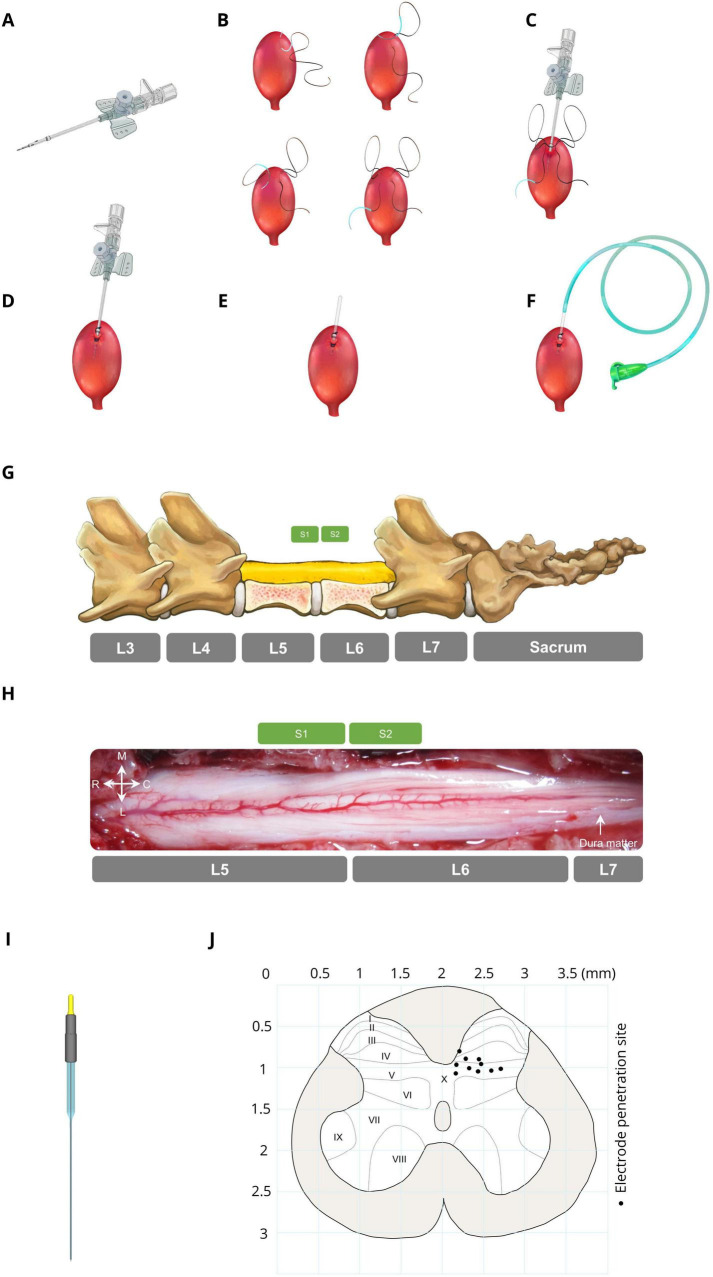
Bladder catheter placement and electrode implantation. **(A)** Modified catheter (a collar for securing the catheter is located 1.1 cm from the top, with 3 drainage holes in this area). **(B)** Applying a purse string suture on the bladder dome. **(C)** Punching and introducing the modified catheter into the bladder dome. **(D)** Fixing the modified catheter by closing the purse-string suture. **(E)** Cutting the extra part of the modified catheter. **(F)** Attaching a 6-Fr catheter to the end of the modified catheter. **(G)** Laminectomy area (L5–L6) and spinal sacral segments relative to the spinal vertebrae in cats. **(H)** Dorsal view of the sacral spinal cord surface in a representative cat. **(I)** Stimulation microelectrode the electrodes were made from epoxy lite-insulated tungsten microelectrodes (length range 61–70 mm, impedance range 50–100 kΩ, shank diameter 125 μm, 15–20° tapered tip, and rounded tip with ∼3-micron diameter at the tip; serial: UE WMEFSEANNF, FHC Inc., Bowdoin, ME, USA). **(J)** Distribution of the electrode positions in the S2 segment (black circles). Each point on the map represents the ISMS stimulation site for each cat.

The bladder was accessed through a suprapubic modified angiocath (Figure 1A) used for injection, drainage, and pressure measurement. The end of a 16G angiocath (Supa, Iran) was cut by 1 cm, and a collar (2 mm in length) was attached to the new tip at 1.1 cm using glow. To avoid sticking the catheter end to the bladder wall, which may cause incomplete draining, three holes (1 mm in diameter) were created on the top of the angiocath. The bladder was exposed via a midline abdominal incision, and a purse-string suture (4–0 silk, Supa, Iran) was applied to the bladder dome (Figure 1B). The modified angiocath was then introduced into the inner space of the bladder by puncturing two distinct layers of the bladder with a sharp hypodermic needle on the bladder dome (Figure 1C), which was secured with a purse-string suture (Figure 1D). Thereafter, the needle of the angiocath was withdrawn (Figure 1E), and the end of the modified angiocath was cut and attached to a 6-Fr catheter (Supa, Iran) (Figure 1F). The abdominal wall and skin were closed in layers with absorbable (Vicryl 3-0, Supa, Iran) and non-absorbable (3-0 nylon, Supa, Iran) sutures, respectively. The suprapubic catheter was connected to a solid-state pressure transducer (MX860, Smiths Medical, UK) and an infusion pump (SN-50C6, Sino Medical-Device Technology Co., China) via a 3-way connector. The bladder pressure was amplified (900×) with a custom-made amplifier (Iran Neural Technology Center, Iran) and sampled at 50 Hz with a 12-bit analog-to-digital converter (Advantech PCI-1711L I/O card, Taiwan).

### 2.4 Electrode implantation

To implant the electrode in the spinal cord to inhibit bladder contraction, initially, the lumbosacral space was identified by touching a valley-like area near the sacrum, and the L4-S1 dorsal vertebral spinous processes were marked on the skin (Figure 1G). The sacral spinal cord segment is usually located under the caudal part of the L5 vertebra and rostral part of the L6 vertebra and varies slightly between different subjects (Shkorbatova et al., 2019; Veshchitskii et al., 2022). An incision was made in the skin, and the underlying muscles were cut. The L5 and L6 dorsal spinous processes were exposed, thinned by a micromotor (Handy 700, Marathon, South Korea), and removed, followed by dorsal laminectomy to expose the end part of the lumbosacral enlargement with its caudal extent (Figure 1H). The dura mater over this area was opened longitudinally, and the cord was covered with warm mineral oil to prevent desiccation during the experiment. After laminectomy, the cat was positioned in a stereotaxic setup (SN–1N, Narishige Group Product, Japan), while the spinal vertebrae (L4 and L7) were clamped rigidly to the frame.

Stimulus pulses were delivered through microelectrodes implanted at the base of the dorsal horn of the S2 spinal cord. The electrodes were made from epoxy lite-insulated tungsten microelectrodes (length range 61–70 mm, impedance range 50–100 kΩ, shank diameter 125 μm, 15–20° tapered tip, and rounded tip with ∼3-micron diameter at the tip; serial: UE WMEFSEANNF, FHC Inc., Bowdoin, ME, USA) (Figure 1I). Each electrode was mounted in a micromanipulator (SM-15, Narishige Group Product, Japan) that controlled the three-dimensional positioning of the electrodes in the lumbosacral spinal cord. A reference electrode was attached to the longissimus muscle of the spinal cord for all stimulation electrodes.

Since bladder contraction suppression is elicited only by stimulation of ventral roots containing parasympathetic preganglionic fibers (de Groat et al., 2014) and the majority of preganglionic neurons (74%) and afferent projections are situated in the S2 segment (Yecies et al., 2018), we identified the S2 segment, positioned approximately beneath the rostral portion of the L6 vertebra (Shkorbatova et al., 2019; Veshchitskii et al., 2022), as the optimal site for bladder suppression. The electrodes were positioned at locations within the base of the dorsal horn approximately 200–750 μm lateral from the midline between 700 and 1,100 μm in depth. Figure 1J [Adapted from the work of Yecies et al. (2018)] represents a cross-sectional view of the S2 spinal cord while each point on the map represents the stimulation site for each cat. In the first isovolumetric trial, the electrode was vertically advanced through the spinal cord dorsoventrally with the standard mapping stimulus (15 s, 20 Hz, 100 μA, 100 μs) to determine the best electrode position for bladder inhibition. The electrode was subsequently removed and reinserted in 200 μm increments in three-dimensional rostrocaudally along S2 spinal segments and/or mediolaterally to an adjacent location where the testing was repeated. The site that suppressed the increase in bladder pressure at the time of rise was selected. The electrode penetration location was estimated via the calibrated bars of the micromanipulators. The standard stimulus was 20 Hz, 100 μA, 100 μs with a duration of 15 s (short duration) or 180 s (long duration). The standard stimulus was chosen as optimal for the stimulation of bladder and EUS-controlled neurons on the basis of earlier studies (Carter et al., 1995; Grill et al., 1999; McCreery et al., 2004; Pikov et al., 2007; Pikov et al., 2020; Tai et al., 2004a).

### 2.5 CMG testing

**FIGURE 2:**
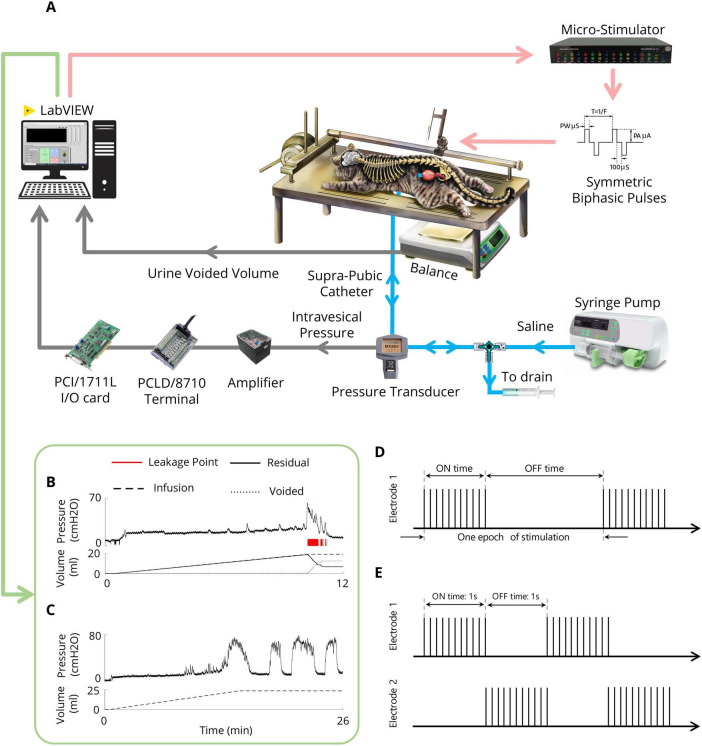
Experimental setup **(A)** Schematic representation of the experimental setup for CMG testing and the ISMS during isovolumetric conditions. The cat is positioned prone on a stereotaxic setup to measure the intravesical pressure, leakage point, and voided volume. A modified suprapubic catheter, pressure transducer, syringe pump, and drain syringe are connected via a 3-way connector. A digital scale beneath the cat measures the voided volume. The pressure signal is amplified and digitized for processing via custom LabVIEW software. In isovolumetric experiments, the digital scale is removed, and the urethral outlet is closed with a 4-Fr catheter. **(B)** An example of the CMG trial for measuring the Vth. **(C)** An example of an isovolumetric trial that shows spontaneous high-amplitude bladder contractions. **(D)** Intermittent stimulation pattern. **(E)** Switching stimulation pattern.

After the recovery period, the bladder was filled with saline via an infusion pump for 30 min to recover the bladder after surgery. For each CMG, the bladder was filled via an infusion pump at a physiological rate (48–120 ml/h) with body temperature (38°C) saline, and the pump was stopped once void or urine leakage occurred. The bladder volume at which leakage occurs and is accompanied by significant bladder contraction refers to the volume threshold (V_*th*_). Two minutes after the pump was turned off, the trial ended. Instant bladder emptying was necessary after each trial to avoid bladder distention. A digital scale (GF-300, A&D Instrument, UK) was positioned under the cat to measure the voided volume. The residual volume could be calculated by continuously subtracting the voided volume from the injected volume. All the experimental setups were implemented in LabVIEW (2017, National Instruments, USA). The experimental setup is depicted in Figure 2A. An example of a CMG trial for measuring V_*th*_ and assessing bladder reflexes is shown in Figure 2B.

### 2.6 Isovolumetric testing

After bladder recovery and initial CMG testing to obtain V_*th*_, the urethral opening was closed with a 4-Fr catheter (Supa, Iran). The bladder was subsequently filled with body temperature (38°C) saline to reach a volume slightly above the V_*th*_ to induce large-amplitude (greater than 30 cmH2O) rhythmic reflex bladder contractions (Tai et al., 2011) (Figure 2C). Isovolumetric trials were separated by 10 min of rest to allow bladder relaxation after stimulation. To test the volume-dependent effect of stimulation, the bladder of one cat was also filled with two times the volume at which rhythmic bladder contractions occur.

### 2.7 Stimulation protocol

A custom-made 16-channel computer-based microstimulator (NeuroStim-4mA, Iran Neural Technology Center, Iran) was used to stimulate the spinal cord. The stimulator generated charge-balanced, symmetric biphasic (cathodic phase first) current pulses with an interphase delay of 100 μs. μm. Different paradigms were employed to evaluate the effects of inhibitory ISMS on bladder activity during isovolumetric conditions, including continuous, intermittent, and switching stimulation (Figures 2D, E). Moreover, the inhibitory effects of stimulus parameters (i.e., pulse amplitude and frequency) were investigated. Additionally, the effects of stimulation parameters during high-volume exercise (2V_*th*_) were investigated.

Intermittent stimulation of the anterior roots S2, S3, and S4 in humans (Liao and Madersbacher, 2019), as well as the pudendal nerve in cats (Boggs et al., 2006a; Tai et al., 2007a), has already been suggested as a potential solution for addressing DSD and enhancing the effectiveness of urinary voiding. In the present study, the effect of intermittent stimulation (Figure 2D) on bladder inhibition was explored by examining different on/off times (0.5 s on, 0.5 s off; 1 s on, 1 s off; 2 s on, 1 s off) with typical stimulation parameters (i.e., 20 Hz, 100 μA, 100 μs) and a stimulation duration of 15 s.

The switching stimulation technique involves asynchronous multielectrode stimulation of the bilateral dorsal horn of the S2 spinal cord (Figure 2E). Intermittent stimulation is alternately applied to the two electrodes that are implanted in the bilateral S2 dorsal horn to inhibit bladder contraction. To increase the effectiveness of the investigation, 15 s of stimulation was initiated when the intravesical pressure exceeded 25 cmH2O from the baseline. This stimulation duration was sufficient to suppress bladder contractions on the basis of earlier works with conditional stimulation of the pudendal nerve (Wenzel et al., 2006).

### 2.8 Nonlinear dynamical analysis

In this work, a recurrence plot (RP) is used to characterize the dynamics of the bladder pressure reflex. A recurrence plot is a method used to visualize the recurrence of dynamic systems. Recurrence is a fundamental property of dynamical systems (Hilborn, 2010; Marwan et al., 2007; Webber and Marwan, 2016). To construct the RP, a symmetrical *N* × *N* array called the recurrence matrix *R*, is calculated as follows:


$$R_{i,j}\,\left(\varepsilon \right)=\Theta \,\left(\varepsilon -\left|{\vec{x}}_{i}-{\vec{x}}_{j}\right|\right)$$

where *N* is the number of intended states $\vec{x}$, ${\vec{x}}_{i}$ is the state of the system at the specific point of *i* in the phase space, Θ(*x*) is the Heaviside function [i.e., Θ(*x*) = 0 if *x* < 0, and Θ(*x*) = 1 otherwise], ε is the threshold distance, and ||⋅|| is a norm. Thus, the recurrence matrix is a matrix consisting of ones and zeros. Since *r*_*i*,*j*_ = *r*_*j*,*i*_, the RP has a black main diagonal line, called the line of identity (LOI).

Small-scale structures of RPs, known as textures, can provide valuable information about the dynamics of a system, which can typically be classified into four classes of single dots, diagonal lines parallel to the LOI, diagonal lines orthogonal to the LOI, and vertical or horizontal lines (Marwan et al., 2007; Webber and Marwan, 2016). The combination of vertical and horizontal lines forms rectangular clusters (Webber and Marwan, 2016). The RPs of each dynamical system have their own topology. For example, RPs related to periodic systems have uncut and long diagonal lines. The vertical distance between these diagonal lines is related to the period of the fluctuations. The chaotic system also has diagonal lines that are shorter than those of periodic systems with certain vertical distances. The vertical distances in chaotic systems are not as regular as those in periodic systems. The RP of the uncorrelated stochastic signal consists of many single black points.

The first step in constructing an RP is an important component of the reconstruction of its phase space of the dynamical system. The phase space of a dynamical system is a space in which all possible states of a system are represented, whereas each possible state of the system corresponds to one unique point in the phase space. A frequently used method for the reconstruction of the phase space from the time series is Taken’s time delay method. In this method, the dynamics of time series (*x*_1_,*x*_2_,…,*x*_*N*_) can be fully captured and embedded in an *m*-dimensional phase space defined by the delay vectors:


$$x_{t}=\left(x_{t},x_{t+\tau },\ldots ,x_{t+\left(m-1\right)\,\tau }\right)$$

where τ is the time delay and where *m* is the embedding dimension (ED). To fully capture the dynamics, an appropriate time delay should be chosen. In this work, we used the function and the FNN methods (Kennel et al., 1992) to estimate the time delay (τ = 2) and embedding dimension (*m* = 4), respectively.

### 2.9 Statistical analysis

**FIGURE 3:**
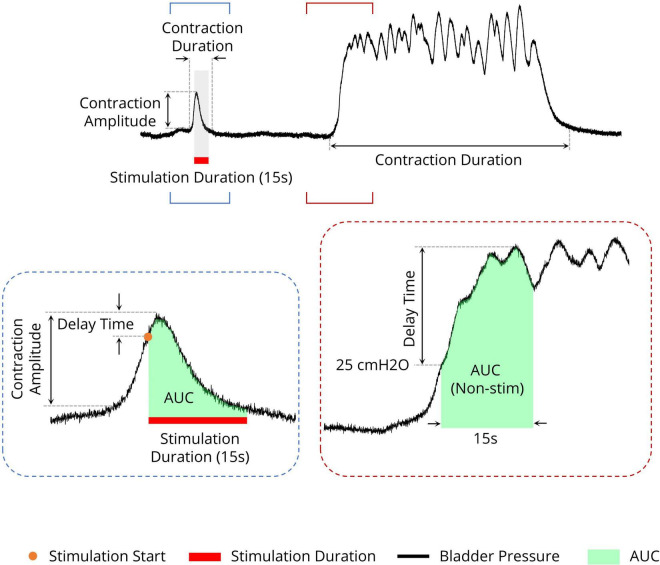
Different measures for evaluating the efficiency of the sacral ISMS on bladder contraction inhibition. The area under the intravesical pressure curve (contraction AUC), maximum intravesical pressure (contraction amplitude), and contraction duration. The ISMS was initiated when the bladder pressure exceeded 25 cmH2O for 15 s (orange circle). The delay time is the duration between the start of stimulation and the maximum intravesical pressure.

To quantify the effects of stimulation during the isovolumetric experiment, different criteria were used, including the area under the intravesical pressure curve (contraction AUC), maximum value of intravesical pressure (contraction amplitude), contraction duration, delay time (the time of the maximum intravesical pressure relative to the start of stimulation), and ICI during the ISMS for a 180-s epoch of stimulation (Figure 3). The measurements were normalized to the measurements obtained prior to stimulation. Moreover, the contraction frequency (i.e., 1/ICI) is used to evaluate the effect of the ISMS on bladder inhibition. When there is complete bladder inhibition or if the duration of inhibition is long, the ICI becomes very long (Wang et al., 2009).

The electrical charge (*Q*) delivered to the tissue per stimulus pulse was also used to evaluate the stimulation protocols and was calculated as follows:


$$Q=\sum_{t=1}^{T}\left|i\,\left(t\right)\right|$$

where *i* represents the current applied during the 15-s stimulation time (*T*).

All acquired data were analyzed via customized MATLAB software (2020b, MathWorks, MA, USA). One-way ANOVA followed by the *post hoc* Tukey–Kramer’s multiple comparison test was used to assess the statistical difference in the results, and *p* < 0.05 (one star), *p* < 0.01 (two stars), and *p* < 0.001 (three stars) indicated a significant difference between groups. Moreover, *p* < 0.05 (one diamond), *p* < 0.01 (two diamonds), and *p* < 0.001 (three diamonds) indicated a significant difference between each group and the control group. Descriptive statistics are given as average ± standard deviation unless otherwise indicated.

## 3 Results

### 3.1 Bladder spontaneous contractions exhibit complex dynamics

**FIGURE 4:**
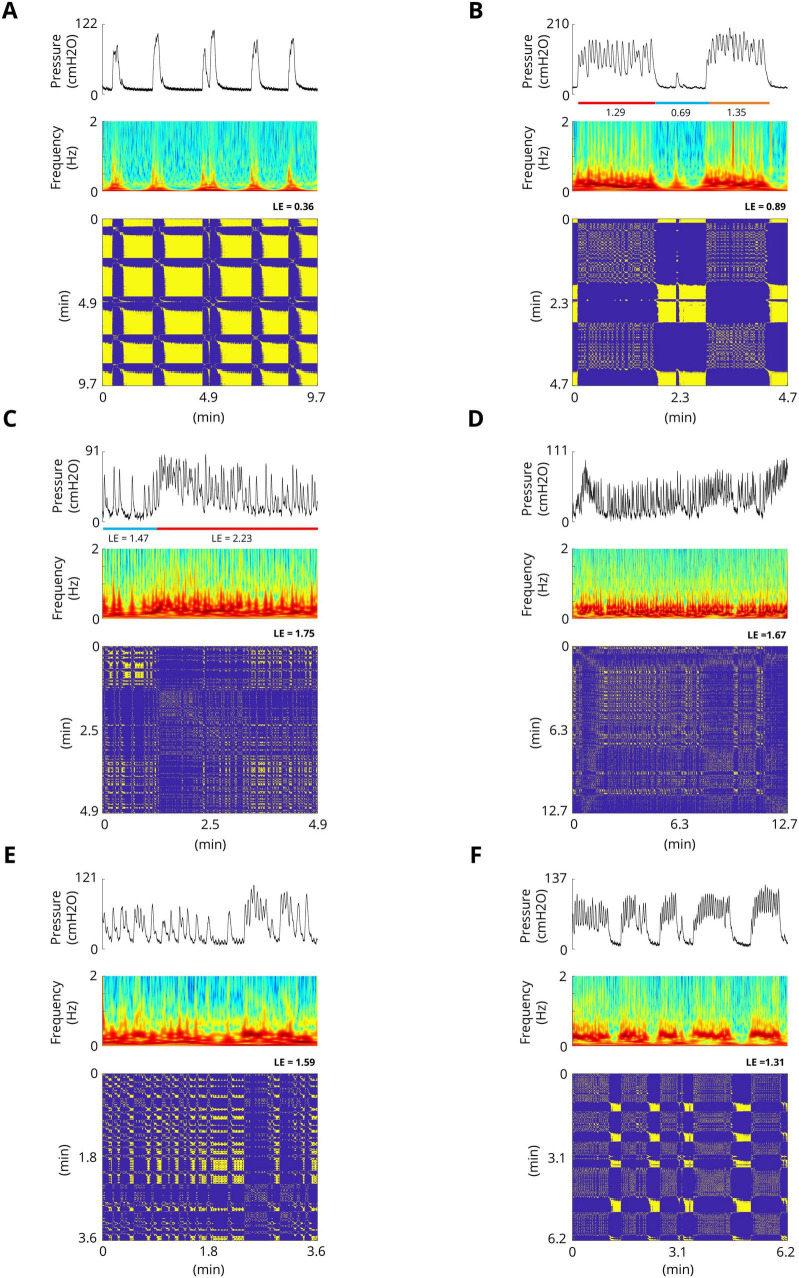
Examples of spontaneous bladder contractions. The recurrence plots for bladder response show different patterns in RPs that are associated with a specific dynamic. **(A)** The RP shows the quasi-periodic of the system. The diagonal lines and checkerboard structures have periodic and quasi-periodic systems. The maximum Lyapunov exponent in this trial is 0.36. **(B)** RP reveals extended clusters of recurrence points, corresponding to many laminar phases (chaos-to-chaos transition). Single, isolated recurrence points indicate that the states of the system are rare and fluctuate strongly. The maximum LE of the first part (red bar) and the third part (orange bar) are 1.29 and 0.89, respectively, whereas the maximum LE of the second part (blue bar) is 0.69, which indicates almost a fixed-point dynamic. **(C)** Chaos-to-chaos intermittency with different LE values. **(D,E)** Chaotic bladder contraction dynamics (single, isolated recurrence points). The maximum Lyapunov exponents are 1.67 **(D)** and 1.59 **(E)**. **(F)** Intermittent chaos.

The results of the experiments show that the bladder response exhibits complex behavior. Figure 4 shows examples of the bladder response, power spectrum of the bladder pressure estimated via the Superlets method (Moca et al., 2021), and RP during isovolumetric conditions. A technique to characterize the dynamics of a system is the RP. The recurrence plots for the bladder response exhibit different typical patterns in RPs that are associated with a specific dynamic of the system. Figure 4A shows the quasi-periodic of the system. Diagonal lines and checkerboard structures linked with periodic and quasi-periodic systems have RPs with diagonal oriented, periodic or quasi-periodic recurrent structures (diagonal lines, checkerboard structures). Single, isolated recurrence points indicates that the states of the system are rare and fluctuate strongly (Figures 4B–F). The results also show that the RP reveals extended clusters of recurrence points, corresponding to many laminar phases (i.e., chaos-to-chaos transition) (Figures 4B, C, E, F). The white areas or bands in the RP indicate abrupt changes in dynamics as well as extreme events (Figures 4A, B). The broadened power spectrum of the bladder pressure confirms the chaotic and intermittence behavior of the bladder dynamics. To characterize the dynamics of the bladder response quantitatively, the Lyapunov exponent (LE) was also computed. The LE is greater during irregular response (Figures 4B–E) than during quasi-periodic behavior (Figure 4A). Figures 4B, C shows that there is chaos-to-chaos intermittency with different LE values. One aspect of bladder reflex dynamics can be categorized as simple phasic contraction (Figure 4A), complex phasic contraction (Figures 4B, F), or complex tonic contraction (Figures 4C–E). The RPs of all the experimental trials can be found in the Supplementary Figures 1–17.

### 3.2 Effect of sacral ISMS inhibition on phasic bladder contraction

**FIGURE 5:**
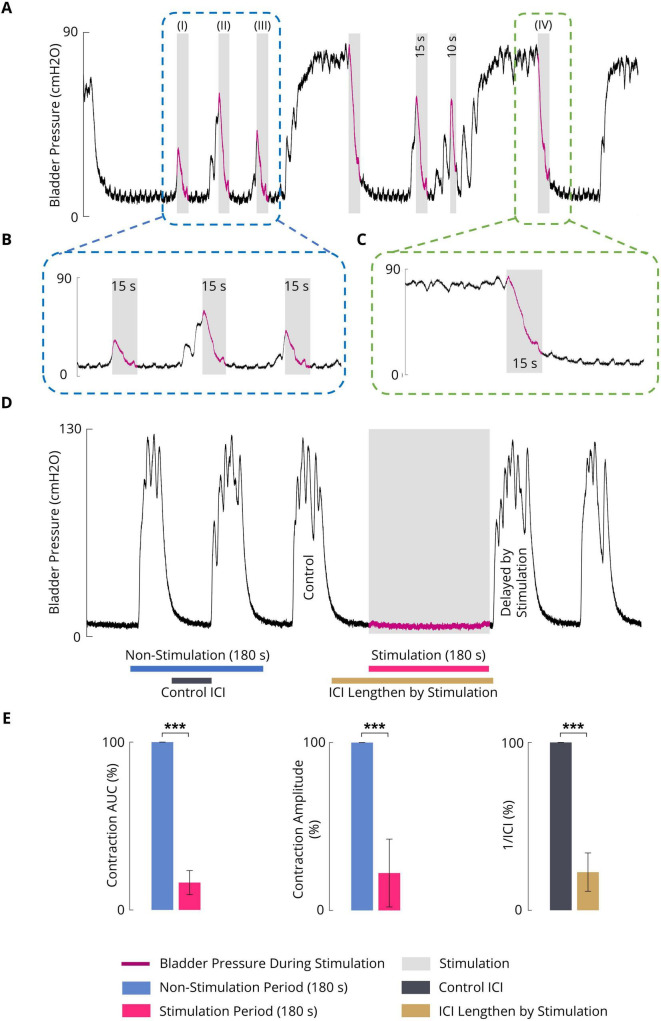
**(A)** An example (cat F1) illustrating the inhibitory effect of the sacral ISMS on phasic bladder contractions. The stimulation was started at the initiation of the contraction (blue box) when the pressure was 12.3 (I), 45.1 (II), and 30.0 cmH2O (III) as well as during the steady-state intravesical pressure (green box) at the high level of 74.5 cmH2O (IV). Stimulation parameters: PA = 100 μA, F = 20 Hz, PW = 100 μs, and duration = 15 s; (I) P_*start*_ = 12.3 cmH2O, P_*max*_ = 26.4 cmH2O, and T_*delay*_ = 1.6 s; (II) P_*start*_ = 45.1 cmH2O, P_*max*_ = 54.3 cmH2O, and T_*delay*_ = 0.9 s; (III) P_*start*_ = 30.0 cmH2O, P_*max*_ = 35.2 cmH2O, and T_*delay*_ = 0.7 s; (IV) P_*start*_ = 74.5 cmH2O and P_*max*_ = 79.1 cmH2O. **(B)** Close-up view of a portion of the plot indicated by the blue box in Figure 5A. **(C)** Close-up view of a portion of the plot indicated by the green box in Figure 5A. **(D)** Typical trial of the long-term inhibitory effect of sacral ISMS inhibition (cat 3) during phasic spontaneous bladder contraction (PA = 100 μA, F = 20 Hz, PW = 100 μs, and stimulation duration = 180 s). **(E)** Results of bladder inhibition by the sacral ISMS on contraction AUC (left), contraction (middle), and 1/ICI (right) during phasic contraction (6 cats). Three stars (***) signify a statistically significance of *p* < 0.001.

Figures 5A–C shows a typical trial of the 15-s ISMS of the spinal S2 dorsal horn with a standard stimulus (20 Hz, 100 μA, 100 μs) during both the initiation of contraction at pressures of 12.3, 45.1, and 30.0 cmH2O (Figure 5A; I–III) and during steady-state intravesical pressure at a high level of 74.5 cmH2O (Figure 5A; IV). It was clearly observed that the ISMS could successfully inhibit bladder contraction. The delay times are 1.6, 0.9, and 0.7 s for the first three stimulation episodes, respectively (Figure 5A; I–III).

To investigate the long-term inhibitory effect of the sacral ISMS, stimulation with standard parameters (i.e., 20 Hz, 100 μA, 100 μs) for 180 s duration was applied to the sacral spinal cord during a typical trial of an experiment with phasic bladder contractions (Figure 5D). The sacral ISMS decreased the contraction AUC and contraction amplitude from 100 and 123.9% cmH2O to 9.8 and 9.7% cmH2O, respectively. Additionally, the ICI was increased from 58 to 231 s. All experimental trials can be seen in the Supplementary Figures 18–24. Overall, the results for 6 cats revealed that compared with the non-stimulation period, the ISMS significantly decreased the 1/ICI, contraction AUC, and contraction amplitude to 22.7 ± 11.4, 6.3 ± 7.2, and 22.3 ± 20.2%, respectively (*p* < 0.001, Figure 5E).

### 3.3 Effect of the ISMS amplitude on the inhibition of phasic bladder contraction

**FIGURE 6:**
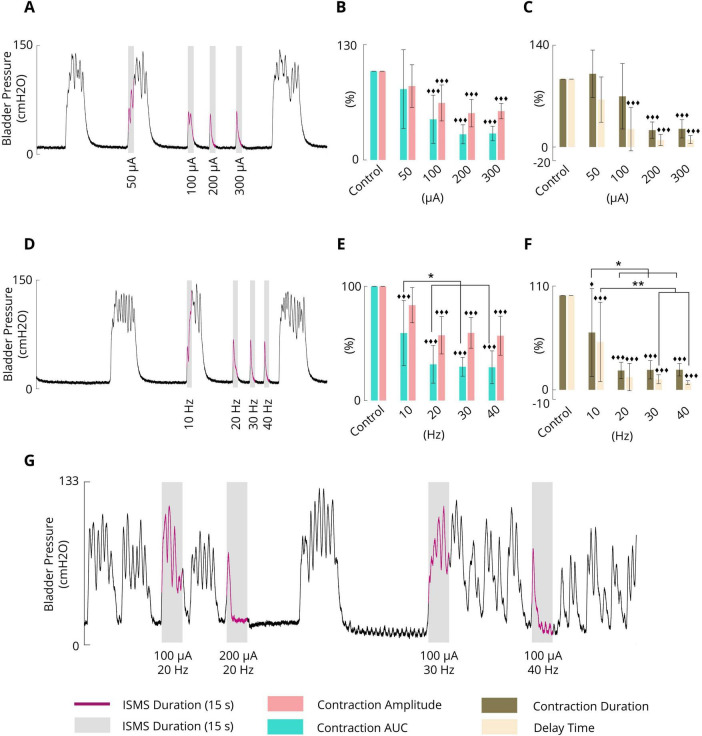
**(A)** Typical trial (cat 3) illustrating the effect of the pulse amplitude (50, 100, 200, and 300 μA) on phasic bladder contraction (F = 20 Hz, PW = 100 μs, and stimulation duration = 15 s). **(B)** Average results obtained by the sacral ISMS over 5 cats with various PAs on the contraction AUC and contraction amplitude. **(C)** Average results obtained by the sacral ISMS over 5 cats with various PAs in terms of contraction duration and delay time. **(D)** Typical trial (cat 3) illustrating the effect of frequency (10, 20, 30 and 40 Hz) on phasic bladder contraction (PA = 100 μA, PW = 100 μs, and duration = 15 s). **(E)** Average contraction AUC and contraction amplitude results obtained by the sacral ISMS at various frequencies. **(F)** The average of results obtained by the sacral ISMS with various frequencies on contraction duration and delay time. **(G)** An example (cat 6) illustrating the effect of the ISMS parameters on phasic bladder contraction when the bladder volume was twice the Vth (PW = 100 μs and stimulation duration = 15 s). Three diamonds (◆◆◆) signify a statistically significance of *p* < 0.001 between each group and the control group, two diamonds (◆◆) for *p* < 0.01, and one diamond (◆) for *p* < 0.05. Two stars (**) signify a statistically significance of *p* < 0.01 between groups and one star (*) for *p* < 0.05.

To evaluate the effect of the varying stimulus amplitude, four different amplitudes, namely, 50, 100, 200, and 300 μA, at a fixed stimulus frequency of 20 Hz and a fixed pulse width of 100 μs were considered. The ISMS was initiated when the bladder pressure exceeded 25 cmH2O for 15 s. A typical trial of the experiment is shown in Figure 6A. All experimental trials can be found in the Supplementary Figures 25–32.

The results for 5 cats revealed that the ISMS with pulse amplitudes of 50, 100, 200, and 300 μA could reduce the contraction AUC to 79.8 ± 44.3, 45.8 ± 27.1, 28.9 ± 10.7, and 29.8 ± 8.2%, respectively, relative to the non-stimulation period (Figure 6B). The results revealed that 50 μA did not significantly reduce the contraction AUC, but 100, 200, and 300 μA significantly reduced the contraction AUC (*p* < 0.001).

Compared with the non-stimulation period, the ISMS with pulse amplitudes of 50, 100, 200, and 300 μA could reduce the contraction amplitude to 83.3 ± 24.1, 64.2 ± 20.3, 52.7 ± 15.5, and 55.1 ± 8.6%, respectively (Figure 6B). The results showed that 50 μA did not significantly reduce the contraction amplitude, but 100, 200, and 300 μA significantly reduced the contraction amplitude (*p* < 0.001). Moreover, the ISMS with pulse amplitudes of 100, 200, and 300 μA reduces the contraction duration by 74.7 ± 48.1, 24.9 ± 12.0, and 26.8 ± 13.4%, respectively, compared with the non-stimulation period, whereas the reduction is significant (*p* < 0.001) for 200 and 300 μA (Figure 6C). Nevertheless, stimulation with 50 μA increased the contraction duration by 107.9 ± 34.9% relative to the non-stimulation period.

Compared with the non-stimulation period, the ISMS with pulse amplitudes of 50, 100, 200, and 300 μA could reduce the delay time to 69.9 ± 33.4, 26.4 ± 32.1, 10.4 ± 8.2, and 10.9 ± 5.9%, respectively (Figure 6C). The results show that 50 μA could not significantly reduce the delay time, but 100, 200, and 300 μA significantly reduced the delay time (*p* < 0.001).

The effectiveness of the stimulation pulse amplitudes on the contraction AUC, contraction amplitude (cmH2O), contraction duration (s), and delay time (s) is also summarized in Table 1. A comparison of the effectiveness of the ISMS amplitudes revealed that those that significantly reduced the outcome compared with non-stimulation did not differ significantly with respect to each other. Overall, the results demonstrated that the ISMS of the spinal S2 dorsal horn with pulse amplitudes of 50, 100, 200, and 300 μA completely suppressed 0, 50, 100, and 100% of the spontaneous phasic bladder contractions, respectively.

**TABLE 1:** Results of ISMS-induced bladder inhibition during phasic contraction.

Experiment	#Trial	Trial name	Contraction AUC (%)	Contraction amplitude (cmH_2_O)	Contraction duration (s)	Delay time (s)	1/ICI (%)
			Mean ± SD	Mean ± SD	Mean ± SD	Mean ± SD	Mean ± SD
Inhibition	7	Control	100.0 ± 0.0	108.8 ± 31.9	–	–	96.5 ± 98.3
		ISMS	16.3 ± 7.2	21.8 ± 21.3	–	–	375.2 ± 219.1
Pulse amplitude	8	Control	100.0 ± 0.0	105.0 ± 30.3	81.2 ± 39.6	13.0 ± 2.7	-
		50 μA	79.8 ± 44.3	85.8 ± 27.3	83.6± 42.4	9.2 ± 4.9	
		100 μA	45.8 ± 27.1	67.6 ± 27.1	53.0 ± 39.7	3.3 ± 4.0	
		200 μA	28.9 ± 10.7	55.8 ± 21.2	17.0 ± 6.4	1.3 ± 0.8	
		300 μA	29.8 ± 8.2	56.3 ± 17.9	18.0 ± 6.1	1.4 ± 0.6	
Frequency	7	Control	100.0 ± 0.0	105.8 ± 29.4	88.8 ± 55.3	13.2 ± 3.1	–
		10 Hz	59.1 ± 28.5	85.7 ± 39.0	59.3 ± 59.4	6.5 ± 5.5	
		20 Hz	31.7 ± 16.4	60.4 ± 27.1	16.5 ± 8.2	1.9 ± 2.2	
		30 Hz	29.6 ± 8.3	61.1 ± 16.7	16.8 ± 8.8	1.5 ± 0.7	
		40 Hz	29.1 ± 14.3	57.0 ± 12.9	16.8 ± 7.5	1.0 ± 0.2	
Intermittent stimulation	6	Control	100.0 ± 0.0	94.5 ± 24.3	82.4 ± 49.2	12.3 ± 3.6	-
		Continuous	30.5 ± 8.4	51.6 ± 15.0	14.5 ± 6.7	2.2 ± 1.9	
		1 s ON/ 1 s OFF	55.2 ± 23.9	60.0 ± 12.0	64.4 ± 59.6	10.3 ± 4.6	
		0.5 s ON/ 0.5 s OFF	61.4 ± 18.6	68.4 ± 25.9	76.3 ± 61.3	7.7 ± 6.9	
		2 s ON/ 1s OFF	36.1 ± 12.4	54.4 ± 17.4	19.9 ± 12.1	2.3 ± 2.2	

### 3.4 Effect of the ISMS frequency on the inhibition of phasic bladder contraction

The frequency dependence of the inhibitory reflex was examined by 15 s ISMS with different frequencies (10, 20, 30, 40 Hz) at a fixed pulse amplitude of 100 μA and a fixed pulse width of 100 μs. The ISMS was started at the instant when the bladder pressure exceeded 25 cmH2O. A typical trial of the experiment is shown in Figure 6D. All trials of the experiment can be found in the Supplementary Figures 33–39.

The results for 6 cats revealed that compared with the non-stimulation period, the ISMS at 10, 20, 30, and 40 Hz significantly decreased the contraction AUC to 59.1 ± 28.5, 31.7 ± 16.4, 29.6 ± 8.3, and 29.1 ± 14.3% (*p* < 0.001, Figure 6E), respectively, and decreased the contraction amplitude to 83.5 ± 15.4, 57.2 ± 16.3, 59.3 ± 13.5, and 56.8 ± 17.1%, whereas the reductions were significant at 20, 30, and 40 Hz (*p* < 0.001, Figure 6E).

The ISMS reduced the contraction duration, respectively, by 60.8 ± 46.6, 20.5 ± 8.7, 21.2 ± 9.7, and 21.3 ± 6.6 (*p* < 0.001 for 20, 30, and 40 Hz and *p* = 0.0168 for 10 Hz, Figure 6F) as well as the delay time by 50.8 ± 42.0, 13.5 ± 14.5, 11.3 ± 4.7, and 7.6 ± 2.0% (*p* < 0.001, Figure 6F).

Table 1 summarizes the results of different frequencies on the contraction AUC (%), contraction amplitude (cmH2O), contraction duration (s), and delay time (s). Comparing the effectiveness of the ISMS frequency with each other, it was found that ISMS frequencies of 20, 30 and 40 Hz, compared with 10 Hz, significantly decreased the contraction AUC (*p* < 0.05), contraction duration (*p* < 0.05), and delay time (*p* < 0.01 for 30 and 40 Hz and *p* = 0.0124 for 20 Hz). However, there were no significant differences between 20, 30, and 40 Hz.

The results of all the trials involving 6 cats revealed that the ISMS at 20, 30, and 40 Hz completely suppressed spontaneous phasic bladder contractions by 28.6, 85.7, 100, and 100% of the trials, respectively.

### 3.5 Effect of bladder volume on ISMS inhibition

In this section, the effect of bladder volume on the inhibition of spontaneous bladder contraction via ISMS is demonstrated. For this purpose, an ISMS with different frequencies and amplitudes was applied during isovolumetric conditions when the bladder volume was 2V_*th*_, which is considered a high volume. Figure 6G shows that the ISMS with standard stimulation parameters (20 Hz, 100 μA, 100 μs, and 15 s) could not suppress bladder contraction when the bladder volume was twice as large as the micturition threshold, whereas stimulation with the 200 μA amplitude and 20 Hz frequency, or 100 μA amplitude with 40 Hz frequency, was effective in inhibiting bladder contraction.

The results of this experiment show that the contraction AUC, contraction amplitude, contraction duration, and delay time were decreased to 30.9%, 70.5 cmH2O, 5.8 s, and 1.2 s, respectively, by the ISMS with a 200 μA amplitude and 20 Hz frequency and to 24.3%, 73.6 cmH2O, 6.2 s, and 0.9 s, respectively, by the ISMS with a 100 μA amplitude and 40 Hz frequency, compared to the non-stimulation bladder contraction which were 100%, 101.8 cmH2O, 22.2 s, and 10.5 s, respectively.

These results indicate that higher stimulation intensity or frequency is required to suppress spontaneous bladder contraction as the bladder volume increases.

### 3.6 Effect of intermittent stimulation on the inhibition of phasic bladder contraction

In this study, the effects of intermittent stimulation (Figure 2D) with standard parameters (20 Hz, 100 μA, 100 μs, 15 s) on the suppression of bladder contraction were examined in three different intermittent stimulation paradigms:

1. Intermittent ISMS with a duty cycle of 50% (0.5 s on and 0.5 s off)
2. Intermittent ISMS with a duty cycle of 50% (1 s on and 1 s off)
3. Intermittent ISMS with a duty cycle of 66% (2 s on and 1 s off)

**FIGURE 7:**
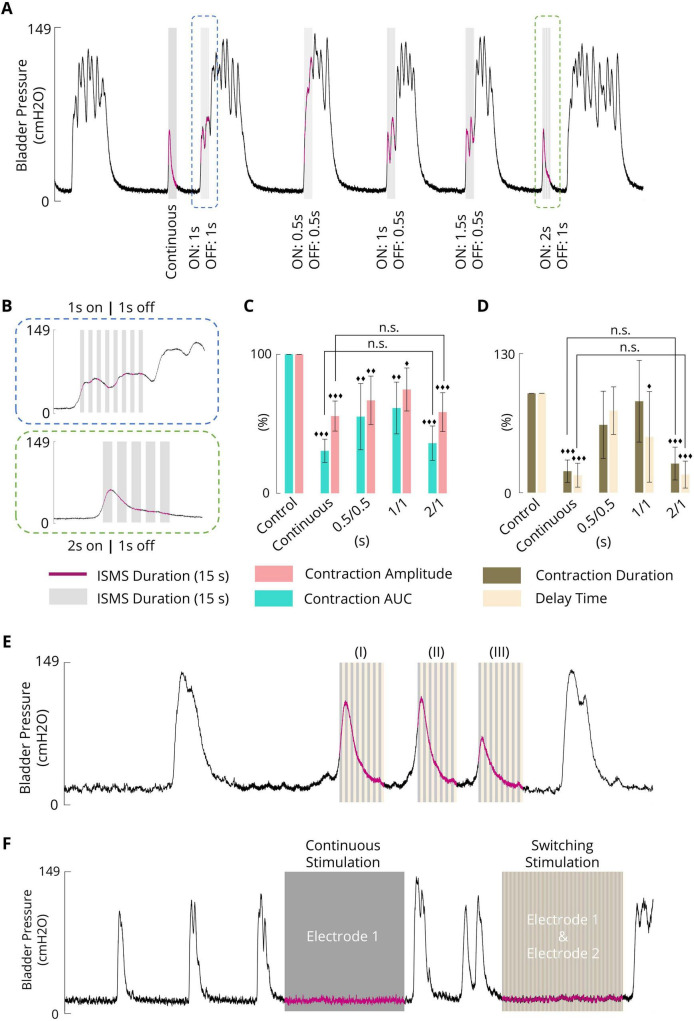
**(A)** A typical trial (cat 3) illustrating the effect of intermittent stimulation with different duty cycles (0.5 s on/0.5 s off, 1 s on/1 s off, and 2 s on/1 s off) on phasic bladder contraction (stimulation parameters: F = 20 Hz, PA = 100 μA, PW = 100 μs, and stimulation duration = 15 s). **(B)** Close-up view of a portion of the plot indicated by the blue and green boxes in Figure 7A. **(C)** The average contraction AUC and contraction amplitude of the results obtained by the sacral ISMS with various on/off times. **(D)** The average of the results obtained by the sacral ISMS with various on/off times for contraction duration and delay time. **(E)** An example (cat 6) illustrating the short-term effect of switching ISMS to suppress phasic bladder contraction. The switching stimulation was started when the bladder pressure was 26.4, 42.5, or 23.1 cmH2O (I, II, and III), respectively. (Stimulation parameters: F = 20 Hz, PA = 100 μA, PW = 100 μs, and stimulation duration = 15 s). **(F)** An example (cat 6) illustrating the long-term effect of switching ISMS to inhibit phasic bladder contraction. A 180-s continuous ISMS was delivered through electrode 1. A 180-s switching ISMS was delivered through electrodes 1 and 2. Three diamonds (◆◆◆) signify a statistically significance of *p* < 0.001 between each group and the control group, two diamonds (◆◆) for *p* < 0.01, and one diamond (◆) for *p* < 0.05.

The ISMS was started at pressures slightly higher than 25 cmH2O. Figure 7A shows a typical experimental trial in which a continuous ISMS and different intermittent ISMSs were applied to suppress spontaneous bladder contraction. Only intermittent stimulation with a duty cycle of 66% (2 s on and 1 s off) suppressed bladder contraction (Figure 7B), similar to continuous stimulation.

The results of the statistical analysis show that intermittent ISMS with a duty cycle of 66% (2 s on and 1 s off) could significantly decrease the contraction AUC (*p* < 0.001), contraction amplitude (*p* < 0.001), contraction duration (*p* < 0.001), and delay time (*p* < 0.001) to 36.1 ± 12.4, 58.4 ± 14.0, 29.3 ± 16.5 and 18.2 ± 13.6%, respectively, compared with the non-stimulation period (Figures 7C, D). However, there was no significant difference between the outcomes of intermittent ISMS with a duty cycle of 66% and those with continuous stimulation (*p* > 0.9) (), whereas the delivered charge to the tissue decreased with intermittent ISMS compared with continuous stimulation. All trials of the experiment can be found in the Supplementary Figures 40–45.

### 3.7 Effect of switching ISMS on phasic bladder contraction

For the switching stimulation paradigm (Figure 2E), two electrodes were implanted in the sacral spinal cord, and the stimulus pulses were delivered to the two electrodes with no interleave time when the duty cycle of stimulation for each electrode was 50% (1 s on, 1 s off) with a 20 Hz frequency, 100 μA amplitude, and 100 μs pulse width. Figure 7E shows an example of the switching ISMS in cat 6. The switching stimulation was started, for a duration of 15 s, when the bladder pressure was 26.4, 42.5, or 23.1 cmH2O (Figure 7E; I–III), respectively. The results show that the contraction AUC, contraction amplitude, contraction duration, and delay time decreased from 100%, 74.8 cmH2O, 23.7 s, and 2.5 s, respectively, which are obtained during uninhibited contraction, to 58.2%, 74.8 cmH2O, 15.4 s, and 2.1 s when the bladder pressure is 26.4 cmH2O, 56.0%, 60.6 cmH2O, 15.0 s, and 1.3 s when the bladder pressure is 42.5 cmH2O, 38.4%, 37.7 cmH2O, 14 s, and 1.3 s when the bladder pressure is 23.1 cmH2O.

Figure 7F shows 180 s of continuous ISMS delivered through electrode one as well as 180 s of switching ISMS delivered through electrodes one and two. Both paradigms increased the duration of spontaneous contractions. The ICI increased from 87.5 s during no stimulation to 209.1 s during continuous stimulation and 210.2 s during switching stimulation.

### 3.8 Inhibitory effect of the sacral ISMS on complex bladder contraction

**FIGURE 8:**
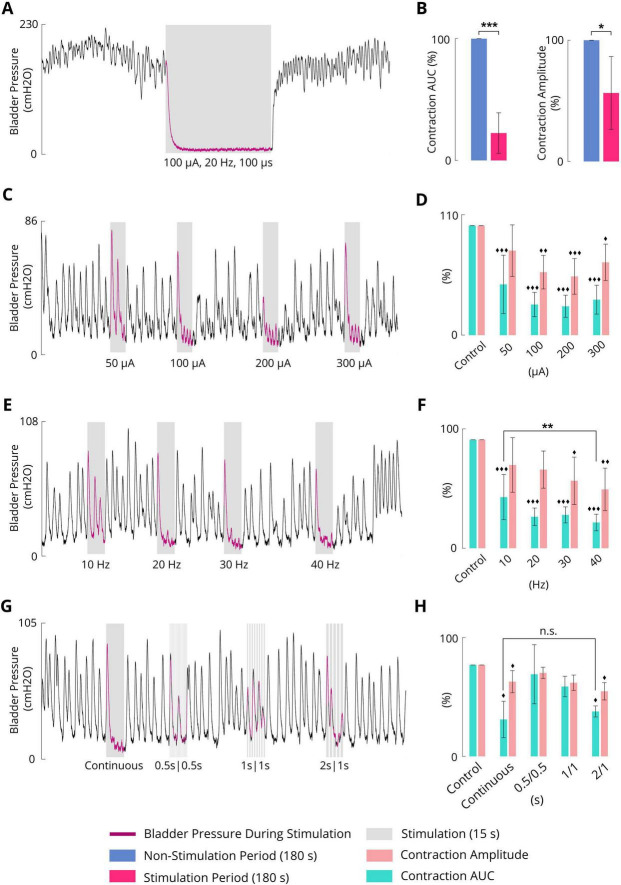
**(A)** Typical inhibition trial (cat 6) for the suppression of complex bladder contraction. Stimulation parameters: PA = 100 μA, F = 20 Hz, PW = 100 μs, and duration = 180 s. **(B)** Results of bladder inhibition by the sacral ISMS on contraction AUC (left) and contraction amplitude (right) (2 cats). **(C)** Typical inhibition trial (cat 4) for the suppression of complex bladder contraction with various PAs. Stimulation parameters: F = 20 Hz, PW = 100 μs, and duration = 15 s. **(D)** Results of bladder inhibition by the sacral ISMS with various PAs on contraction AUC and contraction amplitude (3 cats). **(E)** Typical inhibition trial (cat 4) for the suppression of complex bladder contraction at various frequencies. Stimulation parameters: PA = 100 μA, PW = 100 μs, and duration = 15 s. **(F)** Results of bladder inhibition by the sacral ISMS with various frequencies of contraction AUC and contraction amplitude (3 cats). **(G)** Typical inhibition trial (cat 4) for the suppression of complex bladder contraction with various on/off times. Stimulation parameters: PA = 100 μA, F = 20 Hz, PW = 100 μs, and duration = 15 s. **(H)** Results of bladder inhibition by the sacral ISMS with various on/off times on contraction AUC and contraction amplitude (2 cats). Three diamonds (◆◆◆) signify a statistically significance of *p* < 0.001 between each group and the control group, two diamonds (◆◆) for *p* < 0.01, and one diamond (◆) for *p* < 0.05. Three stars (***) signify a statistically significance of *p* < 0.001 between groups, two stars (**) for *p* < 0.01, and one star (*) for *p* < 0.05.

Figure 8A shows an example of complex bladder contraction while an ISMS with 20 Hz, 100 μA, and 100 μs for a duration of 180 s was applied to the S2 segment almost 3 min after the start of the trial. The sacral ISMS suppressed high-amplitude contractions and decreased the contraction AUC from 100 to 9.8%. Overall, compared with the non-stimulation period, the ISMS significantly decreased the contraction AUC (*p* < 0.001) and contraction amplitude (*p* = 0.0104) to 22.5 ± 16.6 and 55.2 ± 28.2%, respectively (Figure 8B).

To investigate the effects of the stimulation amplitude, the ISMS (20 Hz frequency, 100 μs pulse width, 15 s duration) with different stimulus amplitudes of 50, 100, 200, and 300 μA (Figure 8C) significantly decreased the contraction AUC to 52.3 ± 28.9, 29.5 ± 15.0, 28.9 ± 8.3, and 28.2 ± 6.4%, respectively (*p* < 0.001), as well as significantly decreased the contraction amplitude to 78.0 ± 19.0, 56.4 ± 14.2, 58.5 ± 11.9, and 55.7 ± 18.0%, (*p* < 0.01 for 100 μA, *p* < 0.001 for 200 μA, and *p* = 0.0119 for 300 μA), compared with the non-stimulation period (Figure 8D).

The ISMS at frequencies of 10, 20, 30, and 40 Hz (Figure 8E) significantly decreased the contraction AUC to 52.3 ± 28.9, 29.5 ± 15.0, 28.9 ± 8.3, and 28.2 ± 6.4%, respectively (*p* < 0.001, Figure 8F), as did the contraction amplitude to 78.0 ± 19.0, 56.4 ± 14.2, 58.5 ± 11.9, and 55.7 ± 18.0%, (*p* < 0.05 for 30 Hz and *p* < 0.01 for 40 Hz). Notably, 10 Hz stimulation was less effective than 40 Hz stimulation (*p* < 0.01) in decreasing the contraction AUC (Figure 8F).

An intermittent ISMS with a duty cycle of 66% (2 s on and 1 s off, Figure 8G) was also effective at preventing bladder contraction and decreasing the AUC and amplitude to 49.2 ± 6.1 and 71.2 ± 9.5% (*p* < 0.05, Figure 8H), respectively. The results show that there is no significant difference between the AUC obtained with the intermittent ISMS with a duty cycle of 66% and that obtained with continuous stimulation. However, the contraction amplitude generated by continuous stimulation is lower than that induced by intermittent ISMS (Figure 8H).

All trials of the experiment can be found in the Supplementary Figures 46–67. The effectiveness of different stimulation paradigms during complex contraction is summarized in Table 2.

**TABLE 2:** Results of ISMS-induced bladder inhibition during complex contraction.

Experiment	#Trial	Trial name	Contraction AUC (%)	Contraction amplitude (cmH_2_O)
			Mean ± SD	Mean ± SD
Inhibition	7	Control	100.0 ± 0.0	100.5 ± 18.9
		ISMS	22.5 ± 16.6	55.2 ± 28.2
Pulse amplitude	6	Control	100.0 ± 0.0	88.8 ± 18.0
		50 μA	46.3 ± 26.6	66.2 ± 14.1
		100 μA	27.9 ± 11.1	50.1 ± 11.3
		200 μA	26.3 ± 10.1	46.1 ± 9.2
		300 μA	32.4 ± 13.1	58.3 ± 17.3
Frequency	6	Control	100.0 ± 0.0	89.3 ± 15.1
		10 Hz	47.1 ± 20.8	65.8 ± 13.5
		20 Hz	29.0 ± 8.1	59.5 ± 12.2
		30 Hz	30.7 ± 7.4	62.4 ± 8.4
		40 Hz	23.7 ± 7.6	46.6 ± 16.0
Intermittent stimulation	3	Control	100.0 ± 0.0	83.2 ± 12.1
		Continuous	40.4 ± 19.9	67.9 ± 14.0
		1 s ON/ 1 s OFF	89.9 ± 32.3	75.7 ± 9.0
		0.5 s ON/ 0.5 s OFF	76.4 ± 11.1	66.8 ± 10.6
		2 s ON/ 1 s OFF	49.2 ± 6.1	59.7 ± 14.6

### 3.9 Charge delivery and stimulation paradigm

**FIGURE 9:**
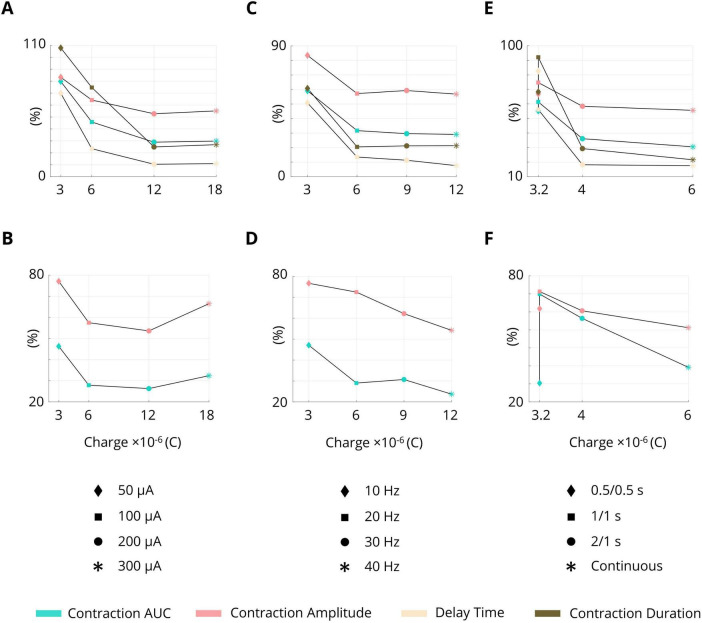
Effects of the stimulation parameters as well as the stimulation paradigms on the inhibition of bladder contraction in terms of charge delivery into the tissue. **(A)** Pulse amplitude during phasic bladder contraction. **(B)** Pulse amplitude during complex tonic bladder contraction. **(C)** Frequency during phasic bladder contraction. **(D)** Frequency during complex tonic bladder contraction. **(E)** Intermittent stimulation during phasic bladder contraction. **(F)** Intermittent stimulation during complex tonic bladder contraction.

The effects of the stimulation parameters as well as the stimulation paradigms in terms of charge delivery into the tissue are summarized in Figure 9. The ISMS with 200 μA (12 × 10^–6^C) provided the best performance for both phasic and complex contractions (Figures 9A, B). During ISMS at 300 μA, leg movement or external urethral sphincter (EUS) contraction was observed in three animals.

Increasing the frequency of stimulation increased the performance. The frequencies of 20 Hz (6 × 10^–6^C), 30 Hz (9 × 10^–6^C), and 40 Hz (12 × 10^–6^C) produced similar performance during phasic bladder contraction (Figure 9C), but the frequency of 40 Hz provided the best performance during complex bladder contraction (12 × 10^–6^C, Figure 9D).

The results also showed that intermittent stimulation with a 66% duty cycle (2 s on, 1 s off) provided almost similar performance to continuous stimulation during phasic bladder contraction (Figure 9E); however, the delivery was charged during intermittent stimulation (4 × 10^–6^C) was 33.3% lower than that during continuous stimulation (6 × 10^–6^C).

### 3.10 Control of the complex dynamics of the spontaneous bladder reflex by ISMS

**FIGURE 10:**
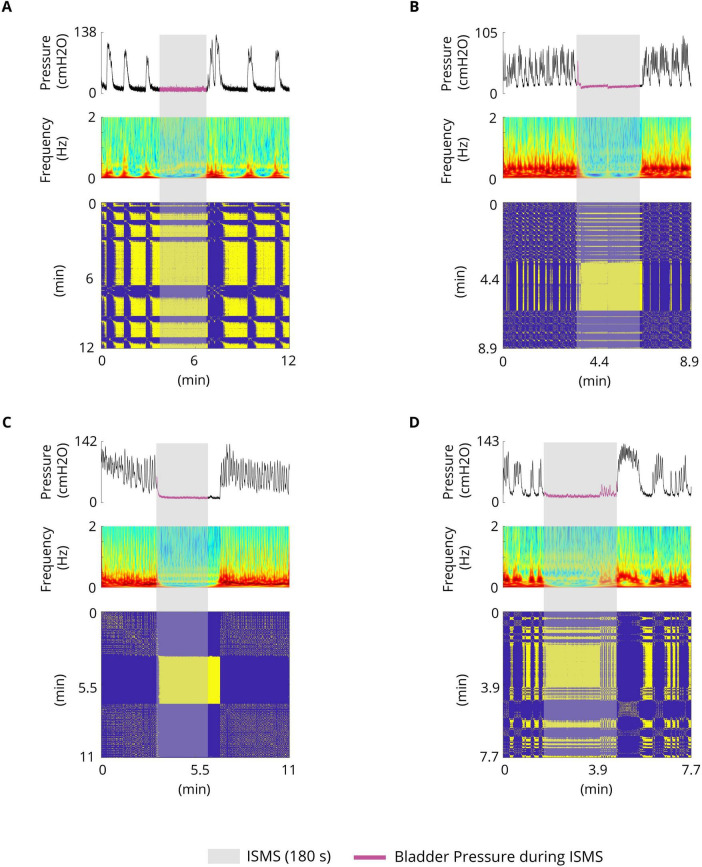
Typical trials illustrating the complex dynamics of the spontaneous bladder reflex and stabilizing (stable equilibrium point) the complex dynamics by ISMS (stimulation parameters: PA = 100 μA, F = 20 Hz, PW = 100 μs, duration = 180 s). **(A)** Simple phasic contraction (cat 1) **(B)** Complex phasic contraction (cat 6) **(C)** Complex tonic contraction (cat 1) **(D)** Complex phasic tonic contraction (cat 4).

In this work, we demonstrated that the spontaneous bladder reflex exhibits complex dynamics, such as mixed mode oscillations, chaotic behavior, and intermittent chaos (Figure 4). The underlying dynamics are not only time-varying; the structure of the dynamics also changes over time, resulting in an uncertain variable structure system. Moreover, the parameters of the system being controlled are not known. To control such uncertain time-varying systems, adaptive control and sophisticated nonlinear control techniques need to be applied. The results of the present study demonstrated that the complex behavior of the spontaneous bladder reflex can be stabilized in a stable equilibrium by ISMS (Figure 10). The quasi-period and the chaotic behavior of the system are effectively controlled to reach the stable equilibrium point.

An important question arises: how is it possible to stabilize such a complex system with an ISMS, given that the system input is constant and has not changed over time? To delve into neural mechanisms of bladder inhibition, a pharmacological study (de Groat, 1976) indicated that a recurrent inhibition in the micturition reflex pathway was mediated at the intraneuronal level prior to the preganglionic neurons. Physiological research (de Groat et al., 2014; Zholudeva and Lane, 2022) has shown that negative bladder interneurons located in gray matter of the spinal cord may be involved in inhibitory mechanisms in the micturition reflex pathways. These interneurons exhibited spontaneous firing (1–30 Hz) with the bladder empty and usually graded reductions in firing with increasing bladder pressure. Additionally, these cells were also synoptically activated by electrical stimulation of afferents in the pudendal space, which is consistent with later research (Tai et al., 2011) on pudendal afferent stimulation, which suggested that bladder inhibition at the spinal level must occur in part by direct inhibitory input to the bladder parasympathetic preganglionic neurons. Hence, the ISMS may activate an inhibitory neural circuit that inhibits bladder contraction.

## 4 Discussion

In this work, the inhibition of spontaneous bladder contraction by ISMS was introduced. Moreover, the effects of the ISMS parameters, intermittent stimulation, and switching stimulation on the inhibition of bladder contraction were investigated. Our findings indicate that ISMS targeting the dorsal horn of the S2 segment effectively suppresses high-amplitude spontaneous contractions. In addition to the contraction AUC and contraction amplitude, we also introduce the contraction duration and delay time for evaluating contraction inhibition.

The results show that spontaneous bladder reflexes exhibit complex dynamics from regular to chaotic. Different transitions from order-to-chaos and chaos-to-chaos (i.e., intermittency) were observed. These intriguing aspects of bladder dynamics, which spontaneously emerge, represent the intrinsic nature of the neural control of the bladder. The design of a control strategy for such nonlinear uncertain time-varying systems is a challenging area of research. The fascinating results of this research are that the ISMS could stabilize the complex behavior of bladder contraction to an equilibrium point (i.e., a constant behavior) and inhibit bladder contraction despite the variability in the bladder pressure and despite what is considered to be the cause of variability. From the results of ISMS inhibition, it can be suggested that the variability observed in measure bladder pressure is caused by the variability in the neural control of the of detrusor muscle. Moreover, in previous reported studies, we can observe the variability in the bladder pressure during CMGs (Hokanson et al., 2021) and isovolumetric conditions (Bruns et al., 2015; McGee et al., 2014; Zhang et al., 2013).

At this point, an important question arises: how can such complex behavior be stabilized to an equilibrium point while sophisticated nonlinear adaptive control should be applied for the control of such systems? Different mechanism at the supraspinal and spinal level may contribute to the continence and urine storage during ISMS. Negative feedback that enables bladder to accommodate larger volumes during filling is related to sympathetic reflex activity (i.e., sympathetic storage reflex). Activation of bladder afferent nerves through stretch of the bladder wall results in increased activity in lumbar sympathetic preganglionic and postganglionic fibers that provide an excitatory input to smooth muscle of the urethra and bladder base and an inhibitory input to smooth muscle in the body of the bladder (de Groat et al., 2014; Zholudeva and Lane, 2022). Since, we implanted ISMS electrode in the sacral spinal cord and sacral sympathetic preganglionic nucleus is located in the lumbar spine (Beckel and Holstege, 2011; de Groat et al., 2014; Zholudeva and Lane, 2022), the ISMS cannot contribute to this negative feedback.

Bladder-to-EUS-to-bladder reflex pathway represents another negative-feedback mechanism in the spinal cord that contribute to urinary continence. Activation of the inhibitory interneurons in the spinal cord suppress reflex bladder activity by inhibiting parasympathetic preganglionic neurons (PGNs) and interneurons on the micturition reflex pathway (de Groat et al., 2014; Zholudeva and Lane, 2022). It may argue that the ISMS could activate this negative feedback and contribute to continence.

Activation of the neurons in the pontine urine storage center (PUSC) promote inhibit micturition by descending inhibitory pathways to the sacral parasympathetic nucleus and activate the EUS motoneurons. In contract, Excitation of the pontine micturition center (PMC) induces relaxation of the bladder neck and the EUS through inhibition of the sympathetic nucleus and Onuf’s nucleus and contracting the bladder through excitation of the parasympathetic nucleus. This process is mediated by the spino-bulbo-spinal reflex, which involves pathways through the bladder, spinal cord (including the dorsal horn and spinal tracts), and brainstem (specifically the pontine micturition center, PMC) (Beckel and Holstege, 2011; de Groat et al., 2014). According to Tai et al. (2011), inhibitory interneurons involved in this reflex, which suppress the transmission of excitatory signals from bladder afferents to the PMC, are located in the sacral spinal segments. It is possible that ISMS inhibit contractions through activation of these interneurons and interfering in the spino-bulbo-spinal reflex. However, more physiological and pharmacological study is needed to be investigated.

Another mechanism is related to urethral sphincter storage reflexes, which serve as negative feedback in the closed-loop control of detrusor contractions. Therefore, we suggest that the ISMS serves as a negative feedback mechanism to inhibit bladder contraction during bladder filling. Our results show that increasing the frequency of the ISMS causes a decrease in bladder pressure, which can be considered a consequence of increasing negative feedback.

The results of this study also revealed that the parameters of the ISMS as well as the stimulation pattern have major effects on the inhibition of bladder contraction. Increasing the intensity of the stimulation (i.e., pulse amplitude and pulse frequency) may not necessarily result in improved inhibition. Moreover, the optimal values of the stimulation intensity or frequency also depend on the bladder volume.

The results of intermittent stimulation show that by proper selection of the duty cycle (i.e., on and off duration), the performance can be obtained via continuous stimulation. However, the rate of charge delivery into tissue using intermittent stimulation is lower than that via continuous stimulation. The switching stimulation paradigm also effectively inhibited spontaneous bladder contraction. The idea behind switching stimulation is to reduce the risk of damage to neural tissue due to chronic stimulation.

The idea behind the selection of the ISMS for the inhibition of bladder contraction was that the ISMS could provide some advantages over peripheral nerve stimulation for bladder control. The grand challenges in peripheral nerve stimulation are nerve displacement, low tissue stability (Liem, 2015; Stuart and Winfree, 2009; Wang et al., 2018a), high-amplitude requirements for neural activation, and unintended efferent fiber activation. ScEs is inherently limited by its lack of electrical field selectivity (Hachmann et al., 2021). Additionally, HFS consumes substantial energy because of its nonphysiological pattern. In contrast, the ISMS addresses many of these issues by leveraging the stability of firm bony structures for implantation, requiring lower stimulation parameters and specifically targeting diverse neural networks within the spinal cord, making it an effective choice for managing bladder dysfunction.

Future works could include investigations of sacral ISMS during non-isovolumetric conditions to evaluate the impact of stimulation on bladder capacity. To achieve this aim, leakage prediction methods such as those used in our previous work (Qasemi et al., 2024) could be used to close the loop of incontinence control. Although most bladder experiments have been conducted during acute and unconscious conditions, it is imperative to conduct bladder experiments during chronic conditions with an awake subject to assess the efficacy in more natural situations. We aimed to address the problems associated with incontinence and OAB; however, owing to the important role of EUS in bladder function, it could be used to evaluate both muscle functions simultaneously in future works. Moreover, in one of our pilot studies, conditionally activating the EUS by stimulating the Onuf nucleus located in the ventral horn of the S1 segment, when pressure exceeded 25 cmH2O, resulted in prevention of leakage and increased bladder capacity compared with the control CMG. Therefore, EUS contraction may offer another viable approach, alongside bladder inhibition, for controlling incontinence, but further study is needed.

In conclusion, this work presents a promising approach for developing neuroprosthesis systems for the control of bladder functions, including voiding and storage. In previous work (Yousefpour and Erfanian, 2021), we demonstrated that ISMS could control bladder voiding and attain a high voiding efficiency of 77.2–100%. Moreover, we have shown that the bladder volume/pressure can be estimated from neural activity recorded directly from spinal cord gray matter neurons (Jabbari and Erfanian, 2019). By combining the method proposed in the current study for the inhibition of bladder contraction with the framework proposed in our previous works (Jabbari and Erfanian, 2019; Yousefpour and Erfanian, 2021), a closed-loop neuroprosthesis system can be developed for restoring bladder functions, which constitutes the future work of this group.

There are several limitations to this study. The experiments in this study were conducted under isovolumetric conditions and is limited to the acute model. Moreover, the measurement during isovolumetric testing is limited to the bladder pressure. Extending the current study to the non-isovolumetric testing, chronic testing using chronically implanted electrodes, and simultaneously measuring the pressure of the bladder and urethra will constitute the future work.

In addition, although the ISMS represents an innovative method for controlling spinal cord functions through the consistent activation of different neural mechanisms by low amounts of charge delivery, the current clinical applicability of the ISMS is restricted. Fabrication and implantation of microwires within the spinal cord present further hurdles for ISMS. Moreover, the act of implanting microwires always poses potential dangers of infection. Nevertheless, advancements in microwire production and insights derived from animal studies on implantable electrodes (Bamford et al., 2017; McCreery et al., 2004; Pikov et al., 2020; Toossi et al., 2016; Toossi et al., 2018) can inform the creation of long-term prosthetic devices for implantation.

## Data Availability

The datasets presented in this study can be found in online repositories. The names of the repository/repositories and accession number(s) can be found below: https://data.mendeley.com/datasets/4xyr64w3dn/1.
