# Supplementary material for: The inhibitory effect of intraspinal microstimulation of the sacral spinal cord on nonlinear bladder reflex dynamics in cats
Source: Front Neurosci. 2025 Feb 3;19:1519377. doi: 10.3389/fnins.2025.1519377 (PMC11830707; doi:10.3389/fnins.2025.1519377)
Supplement: Supplementary file 1 [file Data_Sheet_1.pdf]

Supplementary Material

1 Supplementary Figures

1.1 Contraction dynamics

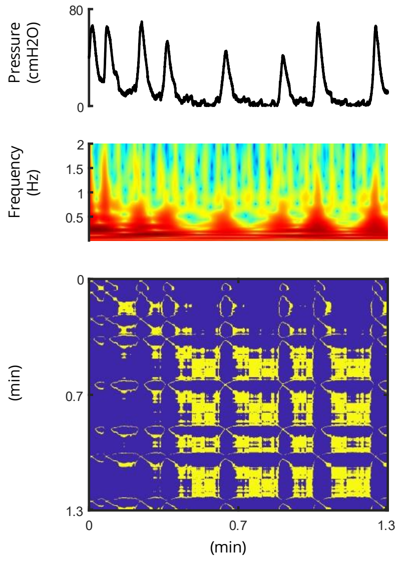

Figure S1. Bladder pressure signal during the isovolumetric condition (top), time-frequency analysis (middle), and recurrence plot of the signal (bottom) in Cat E1, Trial 1, illustrating a simple phasic bladder contraction.

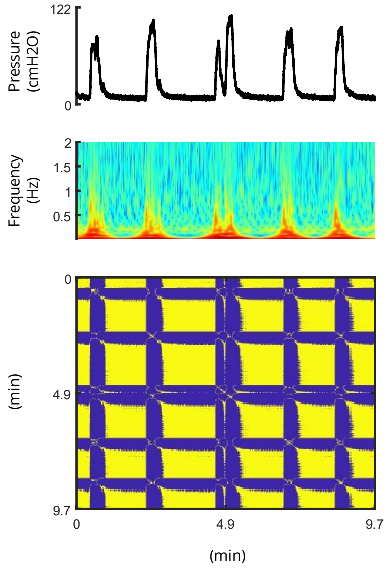

Figure S2. Bladder pressure signal during the isovolumetric condition (top), time-frequency analysis (middle), and recurrence plot of the signal (bottom) in Cat E2, Trial 2, illustrating a simple phasic bladder contraction.

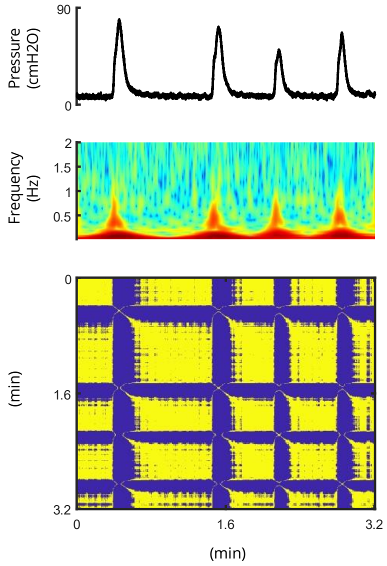

Figure S3. Bladder pressure signal during the isovolumetric condition (top), time-frequency analysis (middle), and recurrence plot of the signal (bottom) in Cat E3, Trial 3, illustrating a simple phasic bladder contraction.

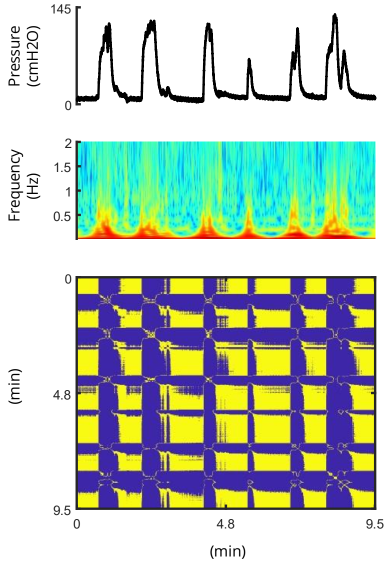

Figure S4. Bladder pressure signal during the isovolumetric condition (top), time-frequency analysis (middle), and recurrence plot of the signal (bottom) in Cat E2, Trial 4, illustrating a simple phasic bladder contraction.

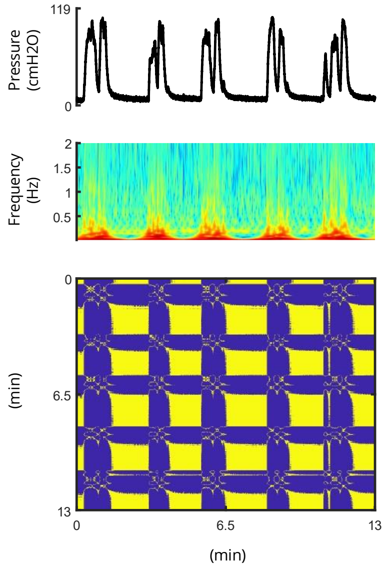

Figure S5. Bladder pressure signal during the isovolumetric condition (top), time-frequency analysis (middle), and recurrence plot of the signal (bottom) in Cat E2, Trial 5, illustrating a complex phasic bladder contraction.

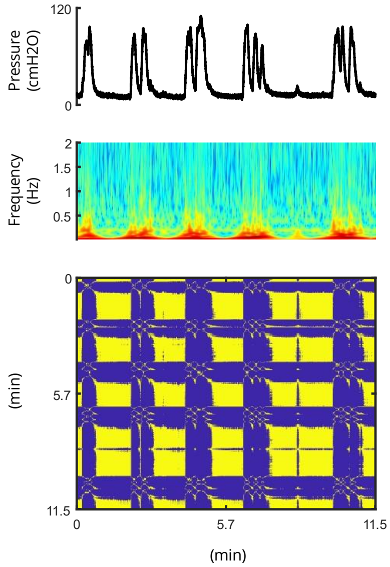

Figure S6. Bladder pressure signal during the isovolumetric condition (top), time-frequency analysis (middle), and recurrence plot of the signal (bottom) in Cat E2, Trial 6, illustrating a complex phasic bladder contraction.

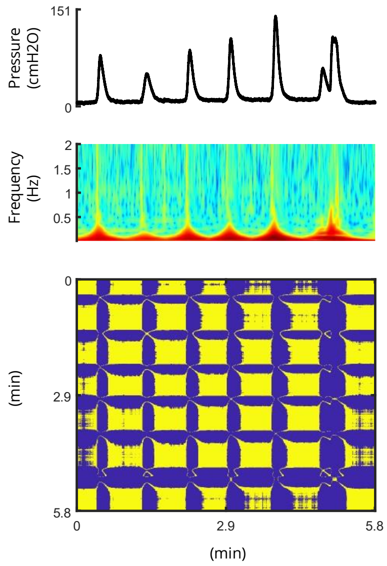

Figure S7. Bladder pressure signal during the isovolumetric condition (top), time-frequency analysis (middle), and recurrence plot of the signal (bottom) in Cat E3, Trial 7, illustrating a simple phasic bladder contraction.

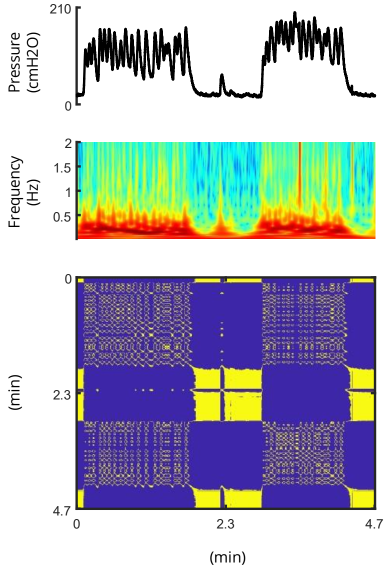

Figure S8. Bladder pressure signal during the isovolumetric condition (top), time-frequency analysis (middle), and recurrence plot of the signal (bottom) in Cat E4, Trial 8, illustrating a complex phasic bladder contraction.

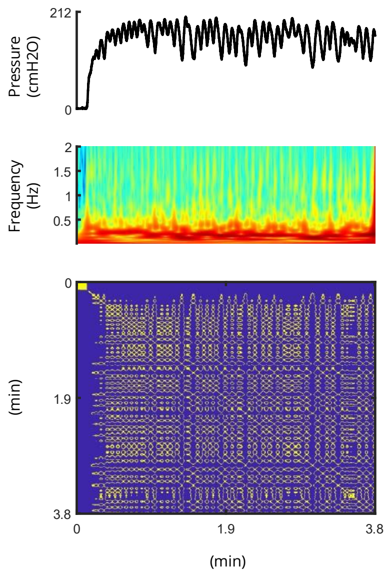

Figure S9. Bladder pressure signal during the isovolumetric condition (top), time-frequency analysis(middle), and recurrence plot of the signal (bottom) in Cat E4, Trial 9, illustrating a complex tonic bladder contraction.

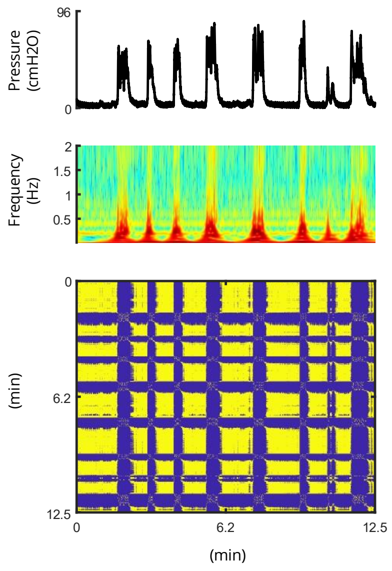

Figure S10. Bladder pressure signal during the isovolumetric condition (top), time-frequency analysis (middle), and recurrence plot of the signal (bottom) in Cat E5, Trial 10, illustrating a complex phasic bladder contraction.

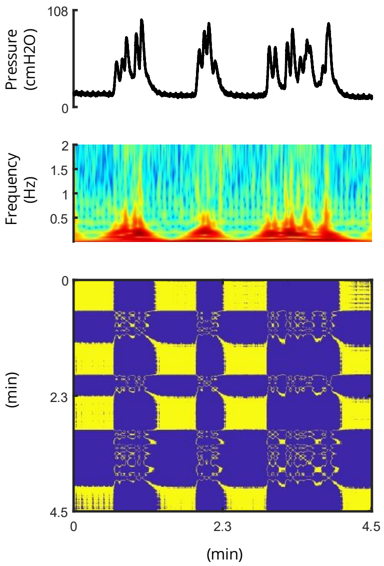

Figure S11. Bladder pressure signal during the isovolumetric condition (top), time-frequency analysis (middle), and recurrence plot of the signal (bottom) in Cat E5, Trial 11, illustrating a complex phasic bladder contraction.

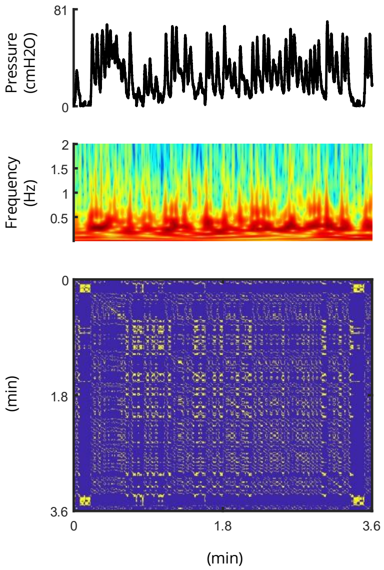

Figure S12. Bladder pressure signal during the isovolumetric condition (top), time-frequency analysis (middle), and recurrence plot of the signal (bottom) in Cat E6, Trial 12, illustrating a complex tonic bladder contraction.

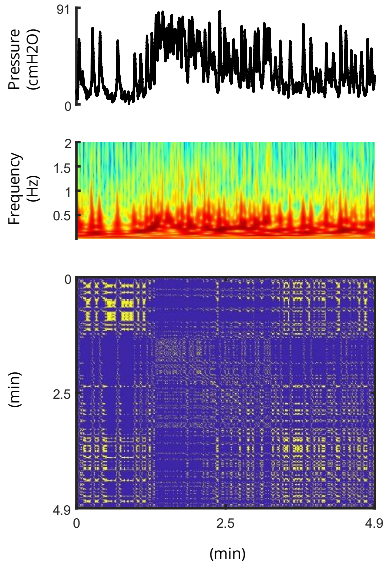

Figure S13. Bladder pressure signal during the isovolumetric condition (top), time-frequency analysis (middle), and recurrence plot of the signal (bottom) in Cat E6, Trial 13, illustrating a complex tonic bladder contraction.

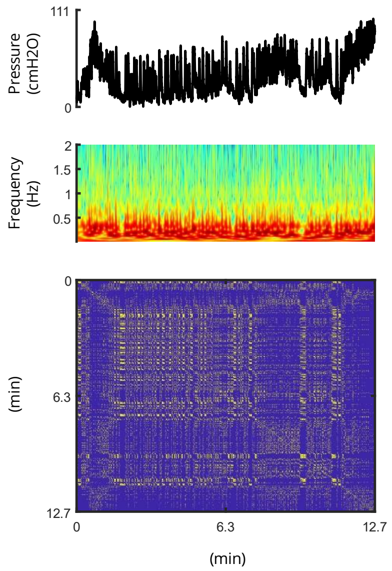

Figure S14. Bladder pressure signal during the isovolumetric condition (top), time-frequency analysis (middle), and recurrence plot of the signal (bottom) in Cat E6, Trial 14, illustrating a complex tonic bladder contraction.

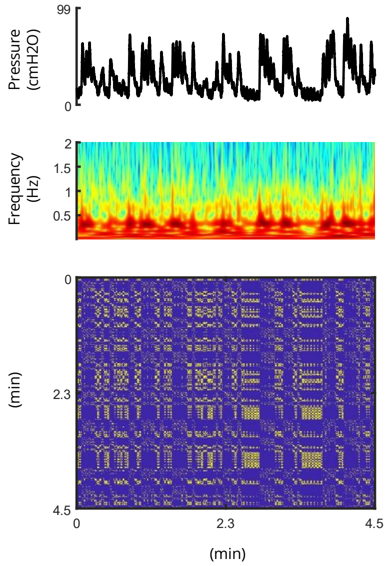

Figure S15. Bladder pressure signal during the isovolumetric condition (top), time-frequency analysis (middle), and recurrence plot of the signal (bottom) in Cat E7, Trial 15, illustrating a complex tonic bladder contraction.

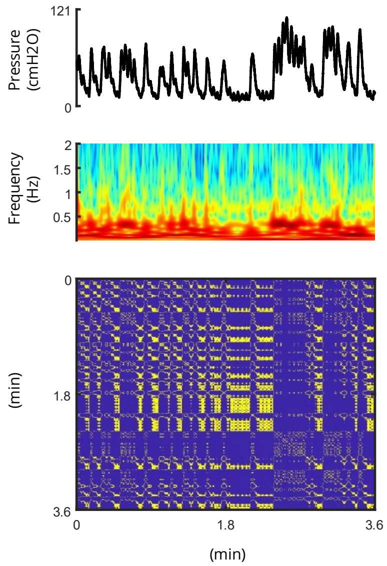

Figure S16. Bladder pressure signal during the isovolumetric condition (top), time-frequency analysis (middle), and recurrence plot of the signal (bottom) in Cat E7, Trial 16, illustrating a complex tonic bladder contraction.

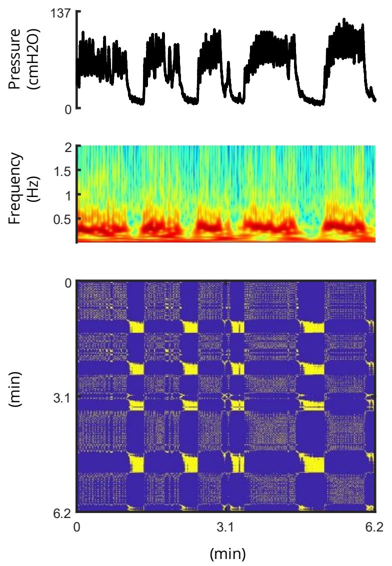

Figure S17. Bladder pressure signal during the isovolumetric condition (top), time-frequency analysis (middle), and recurrence plot of the signal (bottom) in Cat E8, Trial 17, illustrating a complex phasic bladder contraction.

1.2 Bladder phasic contraction inhibition

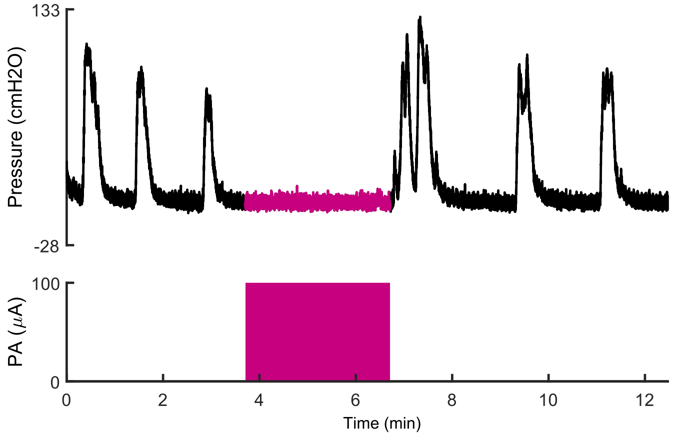

Figure S18. Long-term inhibitory effect of sacral ISMS inhibition (Cat 1, Inhibit 1) during phasic spontaneous bladder contraction (PA = 100 µA, F = 20 Hz, PW = 100 µs, and stimulation duration = 180 s). Bladder pressure signal (top) and stimulation pulse (bottom).

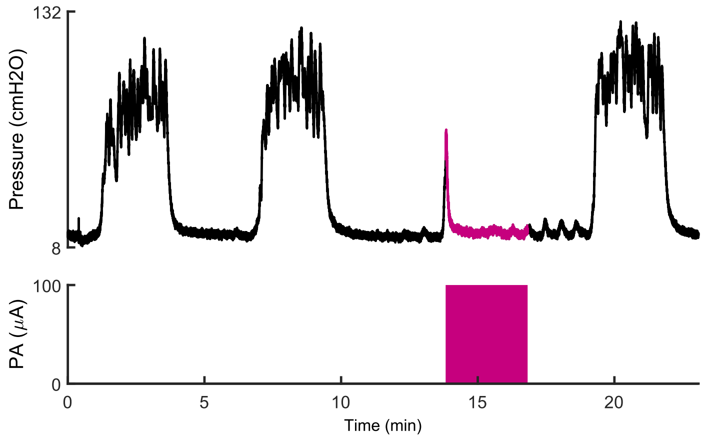

Figure S19. Long-term inhibitory effect of sacral ISMS inhibition (Cat 2, Inhibit 1) during phasic spontaneous bladder contraction (PA = 100 µA, F = 20 Hz, PW = 100 µs, and stimulation duration = 180 s). Bladder pressure signal (top) and stimulation pulse (bottom).

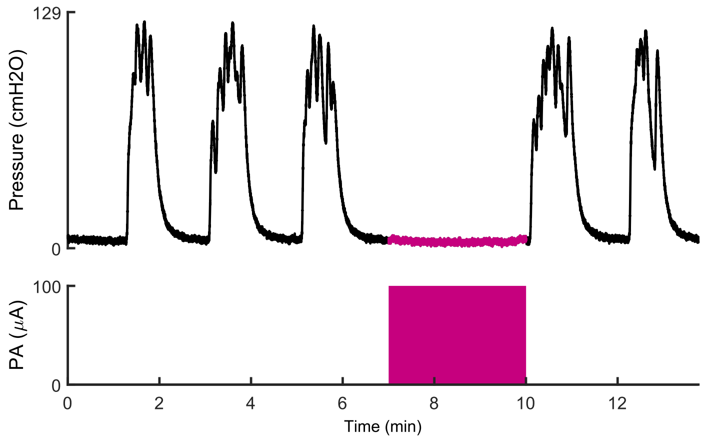

Figure S20. Long-term inhibitory effect of sacral ISMS inhibition (Cat 3, Inhibit 1) during phasic spontaneous bladder contraction (PA = 100 µA, F = 20 Hz, PW = 100 µs, and stimulation duration = 180 s). Bladder pressure signal (top) and stimulation pulse (bottom).

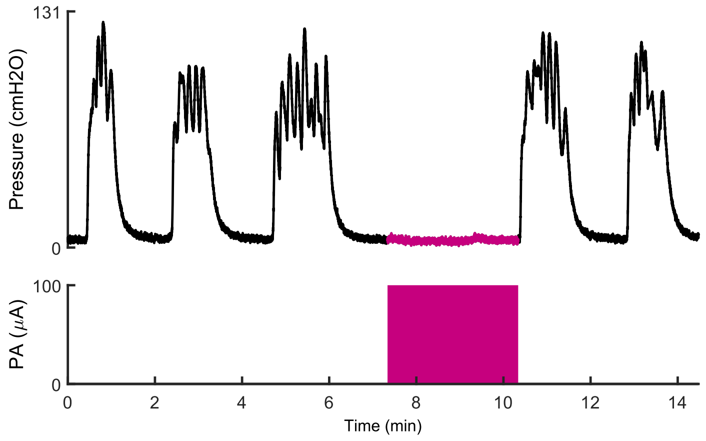

Figure S21. Long-term inhibitory effect of sacral ISMS inhibition (Cat 3, Inhibit 2) during phasic spontaneous bladder contraction (PA = 100 μA, F = 20 Hz, PW = 100 μs, and stimulation duration = 180 s). Bladder pressure signal (top) and stimulation pulse (bottom).

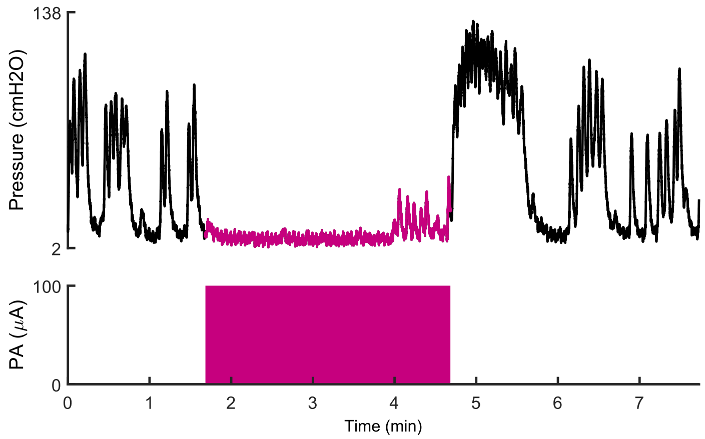

Figure S22. Long-term inhibitory effect of sacral ISMS inhibition (Cat 4, Inhibit 3) during phasic spontaneous bladder contraction (PA = 100 μA, F = 20 Hz, PW = 100 μs, and stimulation duration = 180 s). Bladder pressure signal (top) and stimulation pulse (bottom).

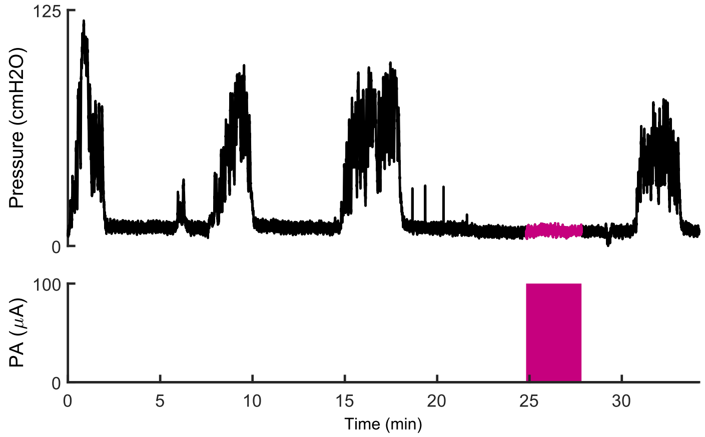

Figure S23. Long-term inhibitory effect of sacral ISMS inhibition (Cat 5, Inhibit 1) during phasic spontaneous bladder contraction (PA = 100 μA, F = 20 Hz, PW = 100 μs, and stimulation duration = 180 s). Bladder pressure signal (top) and stimulation pulse (bottom).

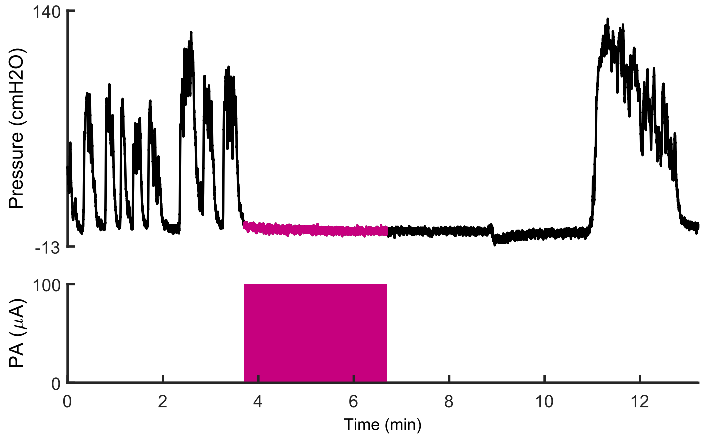

Figure S24. Long-term inhibitory effect of sacral ISMS inhibition (Cat 6, Inhibit 1) during phasic spontaneous bladder contraction (PA = 100 µA, F = 20 Hz, PW = 100 µs, and stimulation duration = 180 s). Bladder pressure signal (top) and stimulation pulse (bottom).

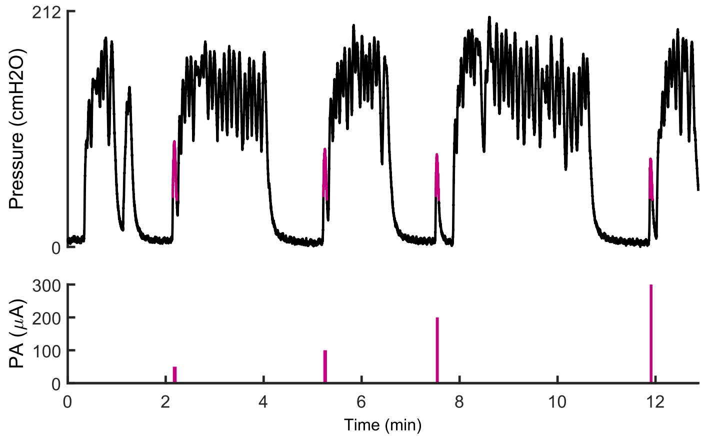

Figure S25. Effect of pulse amplitude of sacral ISMS inhibition (Cat 1, PA1) during phasic spontaneous bladder contraction (F = 20 Hz, PW = 100 µs, and stimulation duration = 15 s). Bladder pressure signal (top) and stimulation pulse (bottom).

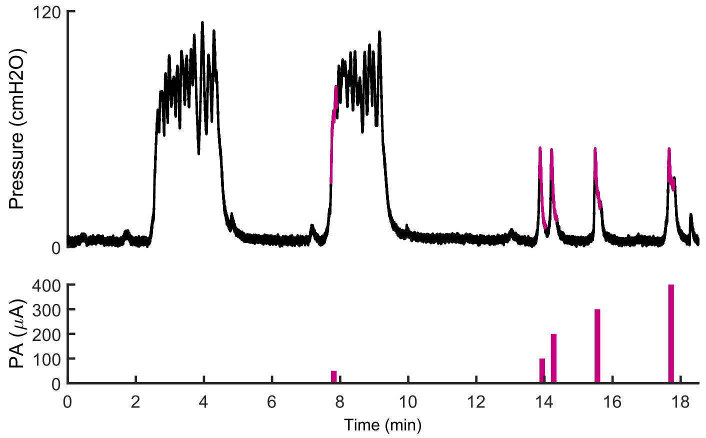

Figure S26. Effect of pulse amplitude of sacral ISMS inhibition (Cat 2, PA1) during phasic spontaneous bladder contraction (F = 20 Hz, PW = 100 µs, and stimulation duration = 15 s). Bladder pressure signal (top) and stimulation pulse (bottom).

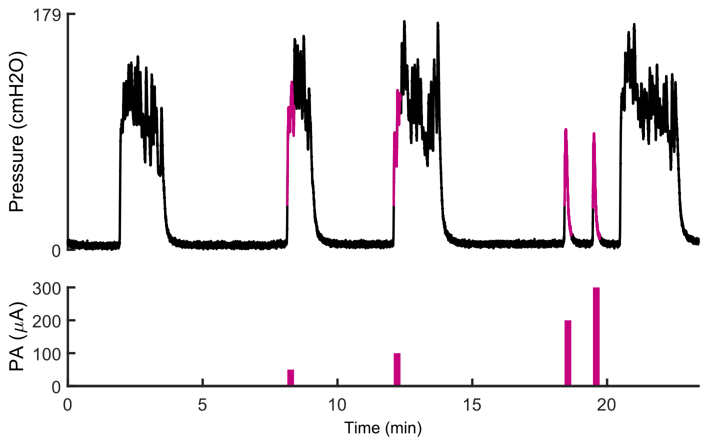

Figure S27. Effect of pulse amplitude of sacral ISMS inhibition (Cat 3, PA0) during phasic spontaneous bladder contraction (F = 20 Hz, PW = 100 µs, and stimulation duration = 15 s). Bladder pressure signal (top) and stimulation pulse (bottom).

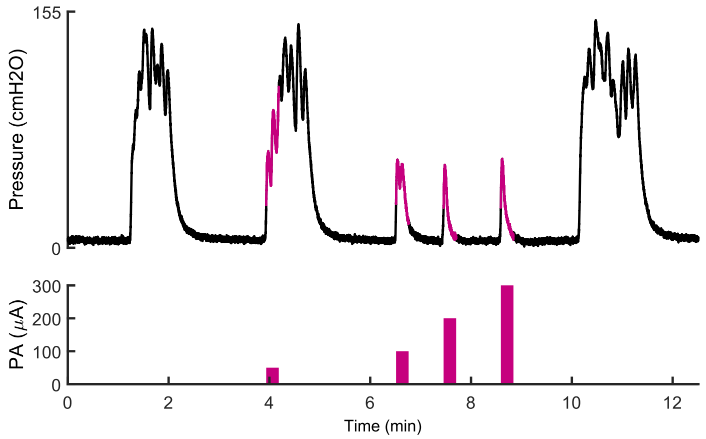

Figure S28. Effect of pulse amplitude of sacral ISMS inhibition (Cat 3, PA1) during phasic spontaneous bladder contraction (F = 20 Hz, PW = 100 µs, and stimulation duration = 15 s). Bladder pressure signal (top) and stimulation pulse (bottom).

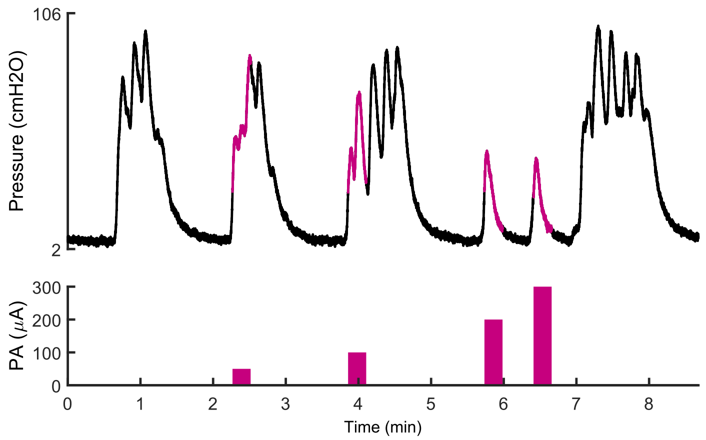

Figure S29. Effect of pulse amplitude of sacral ISMS inhibition (Cat 3, PA2) during phasic spontaneous bladder contraction (F = 20 Hz, PW = 100 μs, and stimulation duration = 15 s). Bladder pressure signal (top) and stimulation pulse (bottom).

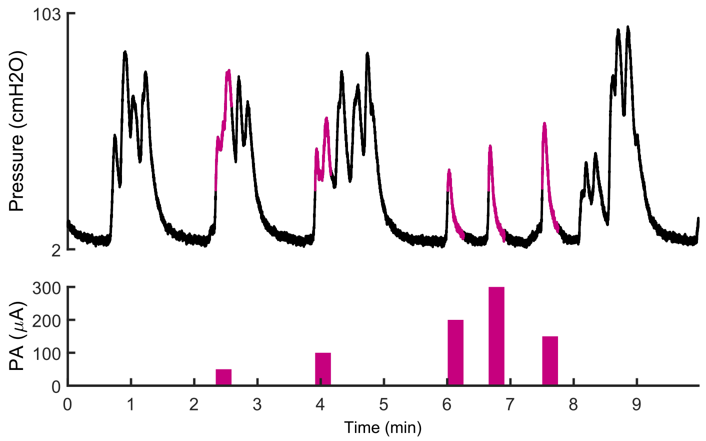

Figure S30. Effect of pulse amplitude of sacral ISMS inhibition (Cat 3, PA3) during phasic spontaneous bladder contraction (F = 20 Hz, PW = 100 μs, and stimulation duration = 15 s). Bladder pressure signal (top) and stimulation pulse (bottom).

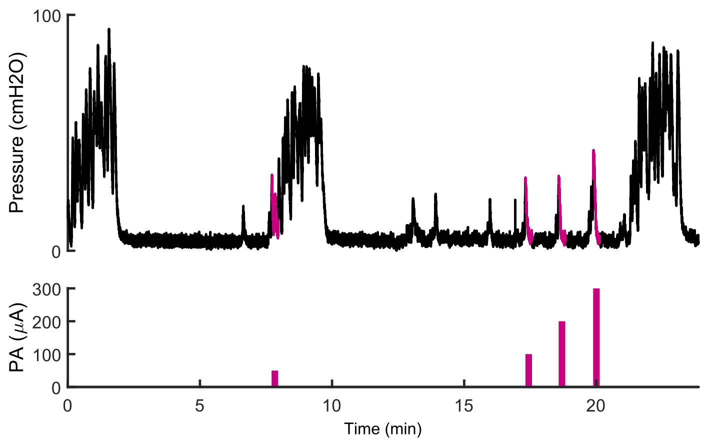

Figure S31. Effect of pulse amplitude of sacral ISMS inhibition (Cat 5, PA1) during phasic spontaneous bladder contraction (F = 20 Hz, PW = 100 μs, and stimulation duration = 15 s). Bladder pressure signal (top) and stimulation pulse (bottom).

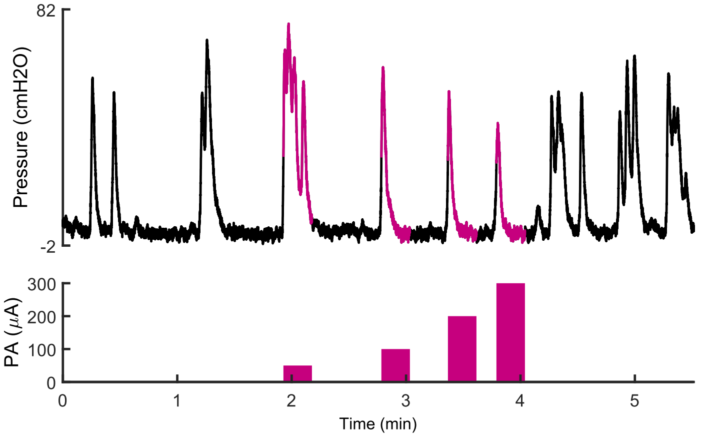

Figure S32. Effect of pulse amplitude of sacral ISMS inhibition (Cat 6, PA1) during phasic spontaneous bladder contraction (F = 20 Hz, PW = 100 μs, and stimulation duration = 15 s). Bladder pressure signal (top) and stimulation pulse (bottom).

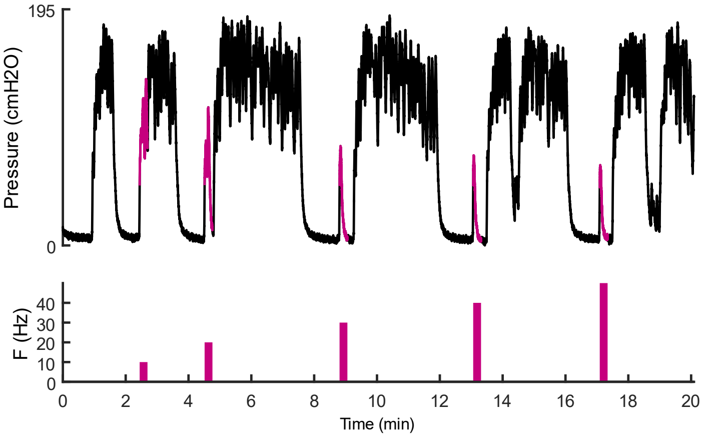

Figure S33. Effect of frequency of sacral ISMS inhibition (Cat 1, F1) during phasic spontaneous bladder contraction (PA = 100 μA, PW = 100 μs, and stimulation duration = 15 s). Bladder pressure signal (top) and stimulation pulse (bottom).

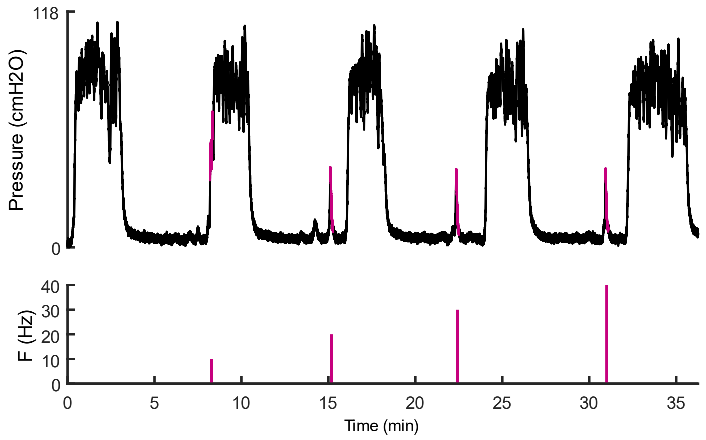

Figure S34. Effect of frequency of sacral ISMS inhibition (Cat 2, F1) during phasic spontaneous bladder contraction (PA = 100 µA, PW = 100 µs, and stimulation duration = 15 s). Bladder pressure signal (top) and stimulation pulse (bottom).

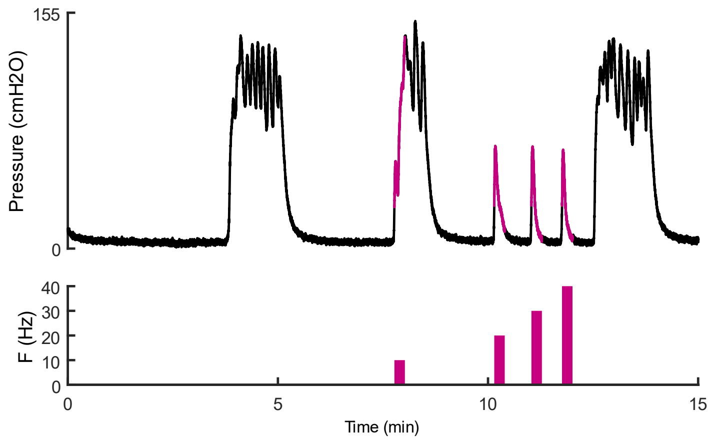

Figure S35. Effect of frequency of sacral ISMS inhibition (Cat 3, F1) during phasic spontaneous bladder contraction (PA = 100 µA, PW = 100 µs, and stimulation duration = 15 s). Bladder pressure signal (top) and stimulation pulse (bottom).

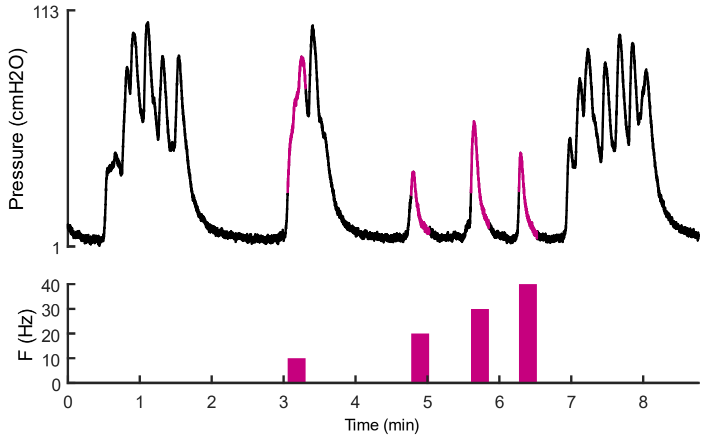

Figure S36. Effect of frequency of sacral ISMS inhibition (Cat 3, F2) during phasic spontaneous bladder contraction (PA = 100 µA, PW = 100 µs, and stimulation duration = 15 s). Bladder pressure signal (top) and stimulation pulse (bottom).

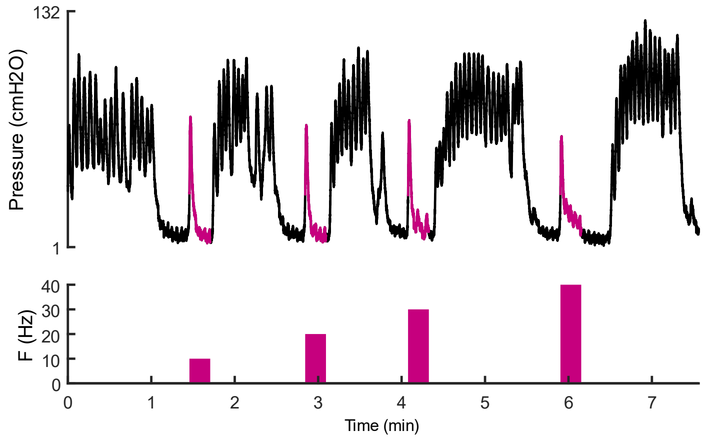

Figure S37. Effect of frequency of sacral ISMS inhibition (Cat 4, F3) during phasic spontaneous bladder contraction (PA = 100 µA, PW = 100 µs, and stimulation duration = 15 s). Bladder pressure signal (top) and stimulation pulse (bottom).

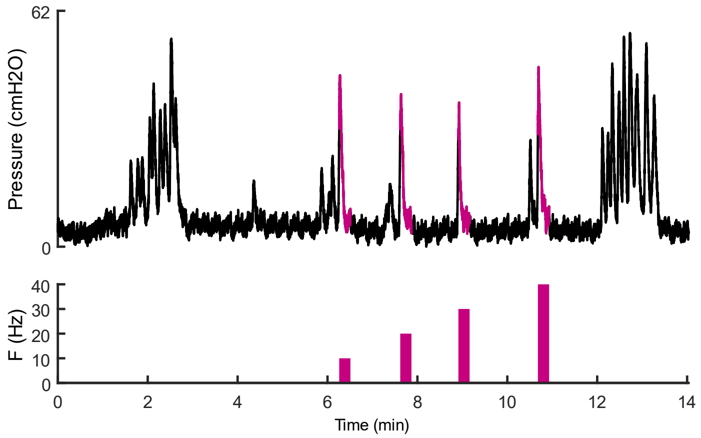

Figure S38. Effect of frequency of sacral ISMS inhibition (Cat 5, F1) during phasic spontaneous bladder contraction (PA = 100 µA, PW = 100 µs, and stimulation duration = 15 s). Bladder pressure signal (top) and stimulation pulse (bottom).

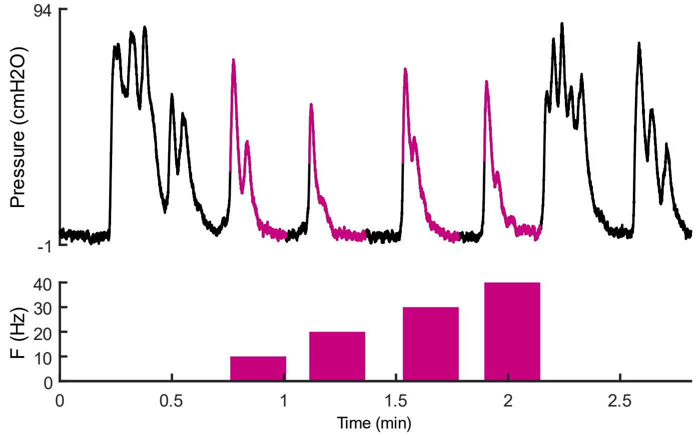

Figure S39. Effect of frequency of sacral ISMS inhibition (Cat 6, F1) during phasic spontaneous bladder contraction (PA = 100 µA, PW = 100 µs, and stimulation duration = 15 s). Bladder pressure signal (top) and stimulation pulse (bottom).

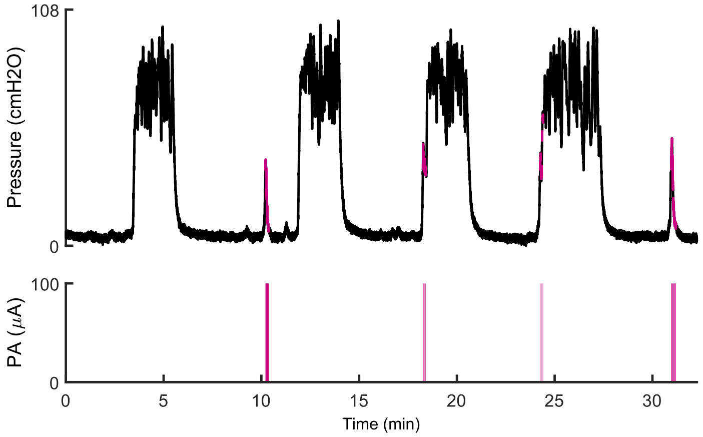

Figure S40. Effect of intermittent pattern of sacral ISMS inhibition (Cat 2, DC1) during phasic spontaneous bladder contraction (PA = 100 µA, F = 20 Hz, PW = 100 µs, and stimulation duration = 15 s). Bladder pressure signal (top) and stimulation pulse (bottom).

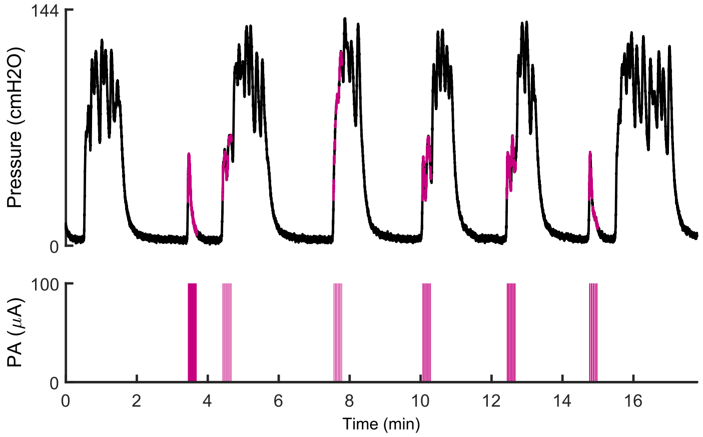

Figure S41. Effect of intermittent pattern of sacral ISMS inhibition (Cat 3, DC1) during phasic spontaneous bladder contraction (PA = 100 µA, F = 20 Hz, PW = 100 µs, and stimulation duration = 15 s). Bladder pressure signal (top) and stimulation pulse (bottom).

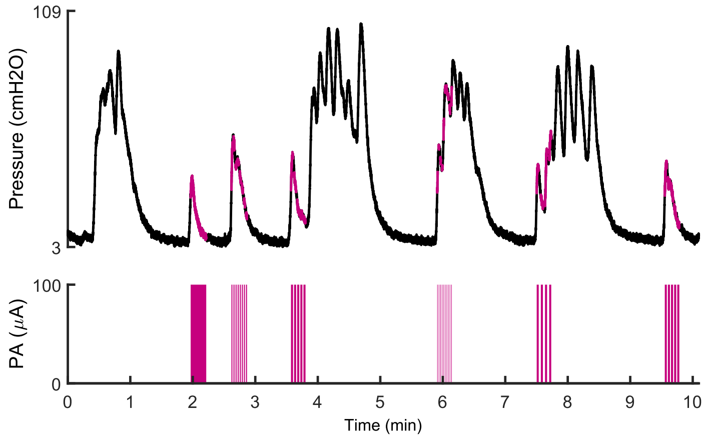

Figure S42. Effect of intermittent pattern of sacral ISMS inhibition (Cat 3, DC2) during phasic spontaneous bladder contraction (PA = 100 µA, F = 20 Hz, PW = 100 µs, and stimulation duration = 15 s). Bladder pressure signal (top) and stimulation pulse (bottom).

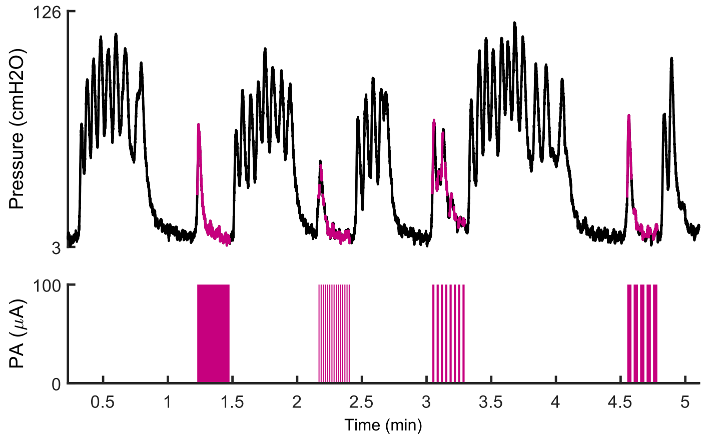

Figure S43. Effect of intermittent pattern of sacral ISMS inhibition (Cat 4, DC3) during phasic spontaneous bladder contraction (PA = 100 µA, F = 20 Hz, PW = 100 µs, and stimulation duration = 15 s). Bladder pressure signal (top) and stimulation pulse (bottom).

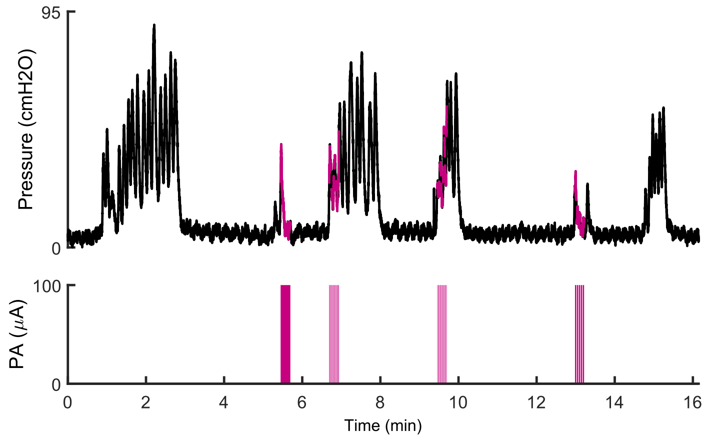

Figure S44. Effect of intermittent pattern of sacral ISMS inhibition (Cat 5, DC1) during phasic spontaneous bladder contraction (PA = 100 µA, F = 20 Hz, PW = 100 µs, and stimulation duration = 15 s). Bladder pressure signal (top) and stimulation pulse (bottom).

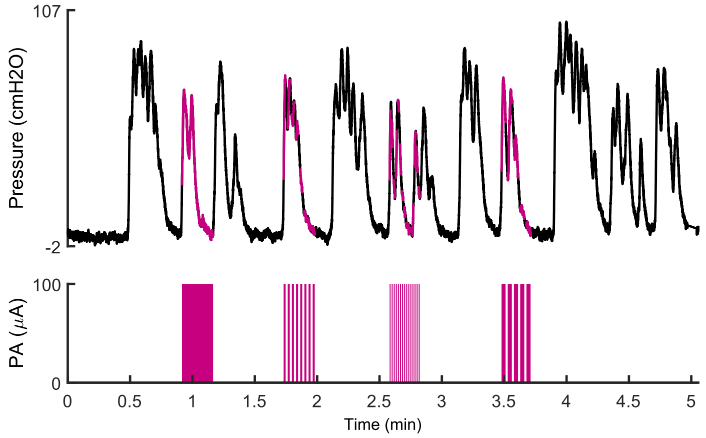

Figure S45. Effect of intermittent pattern of sacral ISMS inhibition (Cat 6, DC1) during phasic spontaneous bladder contraction (PA = 100 µA, F = 20 Hz, PW = 100 µs, and stimulation duration = 15 s). Bladder pressure signal (top) and stimulation pulse (bottom).

1.3 Bladder complex contraction inhibition

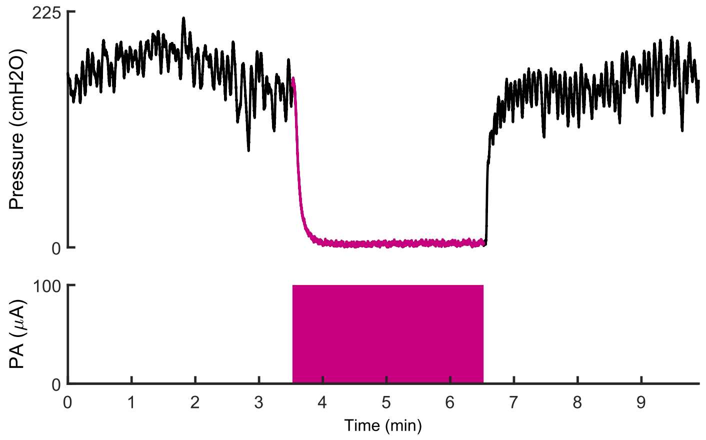

Figure S46. Long-term inhibitory effect of sacral ISMS inhibition (Cat 1, Inhibit 2) during complex spontaneous bladder contraction (PA = 100 μA, F = 20 Hz, PW = 100 μs, and stimulation duration = 180 s). Bladder pressure signal (top) and stimulation pulse (bottom).

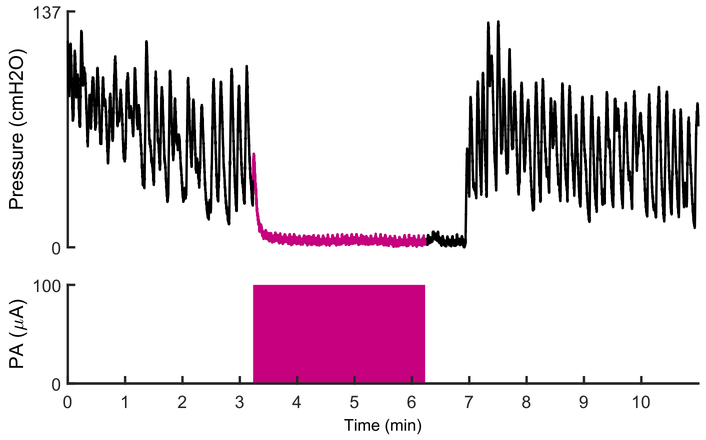

Figure S47. Long-term inhibitory effect of sacral ISMS inhibition (Cat 1, Inhibit 3) during complex spontaneous bladder contraction (PA = 100 μA, F = 20 Hz, PW = 100 μs, and stimulation duration = 180 s). Bladder pressure signal (top) and stimulation pulse (bottom).

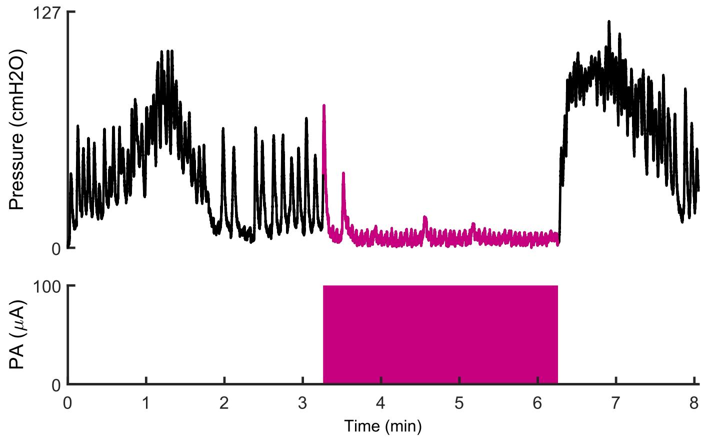

Figure S48. Long-term inhibitory effect of sacral ISMS inhibition (Cat 4, Inhibit 1) during complex spontaneous bladder contraction (PA = 100 µA, F = 20 Hz, PW = 100 µs, and stimulation duration = 180 s). Bladder pressure signal (top) and stimulation pulse (bottom).

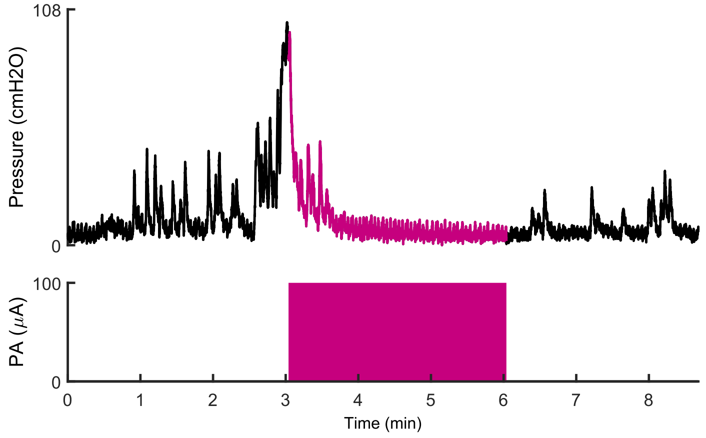

Figure S49. Long-term inhibitory effect of sacral ISMS inhibition (Cat 4, Inhibit 2) during complex spontaneous bladder contraction (PA = 100 µA, F = 20 Hz, PW = 100 µs, and stimulation duration = 180 s). Bladder pressure signal (top) and stimulation pulse (bottom).

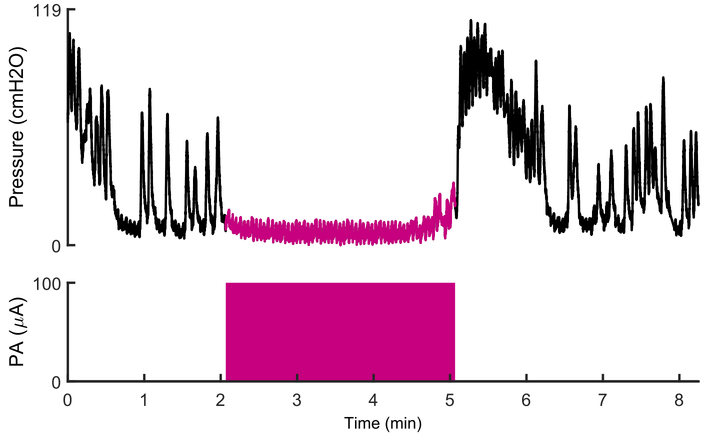

Figure S50. Long-term inhibitory effect of sacral ISMS inhibition (Cat 4, Inhibit 4) during complex spontaneous bladder contraction (PA = 100 µA, F = 20 Hz, PW = 100 µs, and stimulation duration = 180 s). Bladder pressure signal (top) and stimulation pulse (bottom).

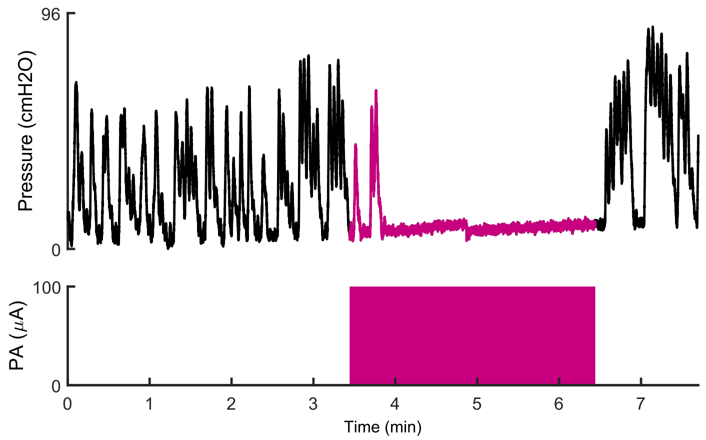

Figure S51. Long-term inhibitory effect of sacral ISMS inhibition (Cat 6, Inhibit 2) during complex spontaneous bladder contraction (PA = 100 μA, F = 20 Hz, PW = 100 μs, and stimulation duration = 180 s). Bladder pressure signal (top) and stimulation pulse (bottom).

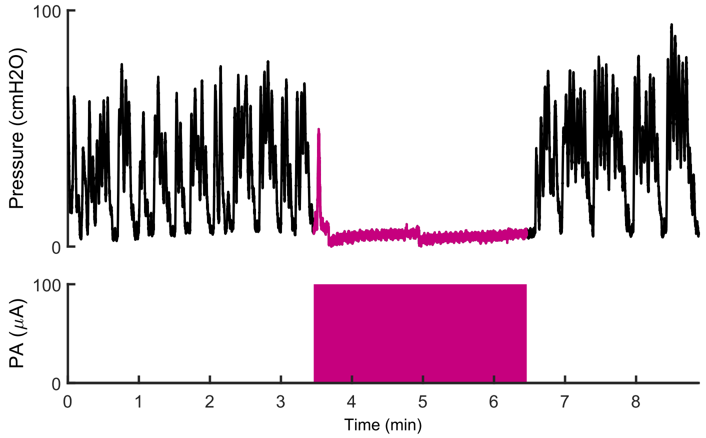

Figure S52. Long-term inhibitory effect of sacral ISMS inhibition (Cat 6, Inhibit 3) during complex spontaneous bladder contraction (PA = 100 μA, F = 20 Hz, PW = 100 μs, and stimulation duration = 180 s). Bladder pressure signal (top) and stimulation pulse (bottom).

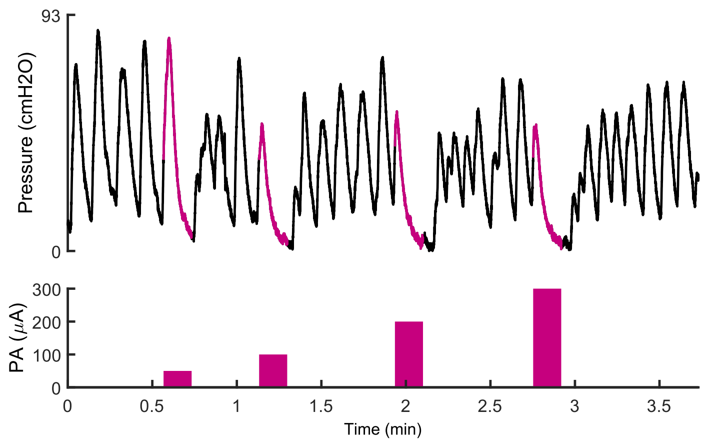

Figure S53. Effect of pulse amplitude of sacral ISMS inhibition (Cat 1, PA2) during complex spontaneous bladder contraction (F = 20 Hz, PW = 100 μs, and stimulation duration = 15 s). Bladder pressure signal (top) and stimulation pulse (bottom).

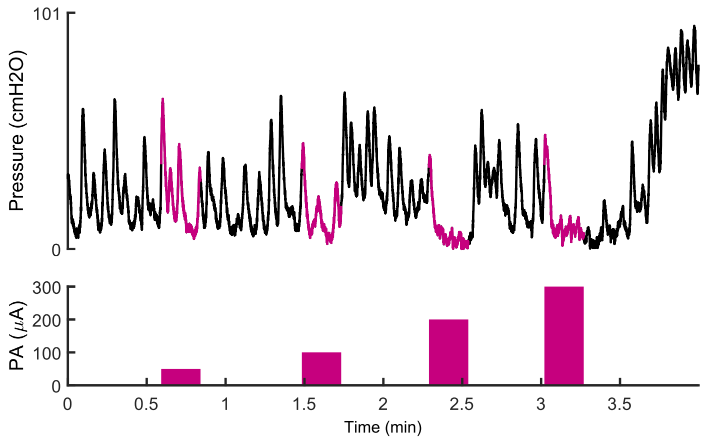

Figure S54. Effect of pulse amplitude of sacral ISMS inhibition (Cat 4, PA1) during complex spontaneous bladder contraction (F = 20 Hz, PW = 100 μs, and stimulation duration = 15 s). Bladder pressure signal (top) and stimulation pulse (bottom).

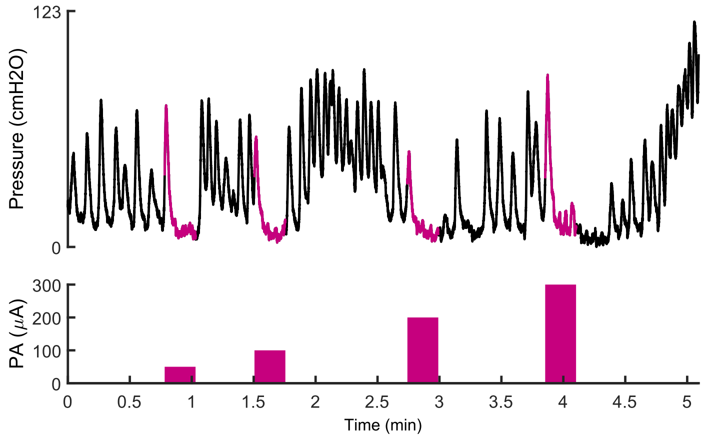

Figure S55. Effect of pulse amplitude of sacral ISMS inhibition (Cat 4, PA2) during complex spontaneous bladder contraction (F = 20 Hz, PW = 100 µs, and stimulation duration = 15 s). Bladder pressure signal (top) and stimulation pulse (bottom).

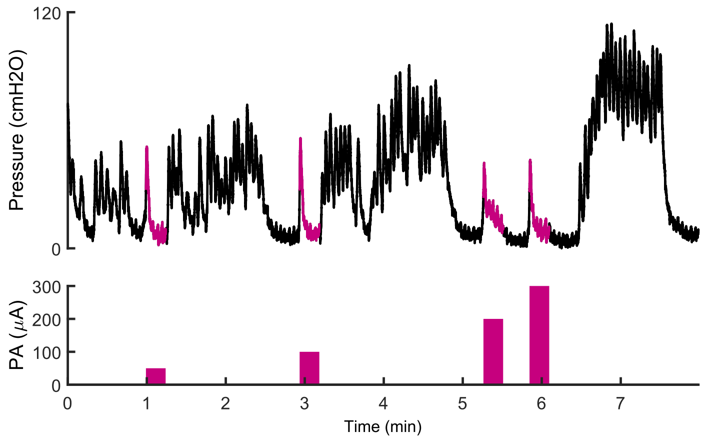

Figure S56. Effect of pulse amplitude of sacral ISMS inhibition (Cat 4, PA3) during complex spontaneous bladder contraction (F = 20 Hz, PW = 100 µs, and stimulation duration = 15 s). Bladder pressure signal (top) and stimulation pulse (bottom).

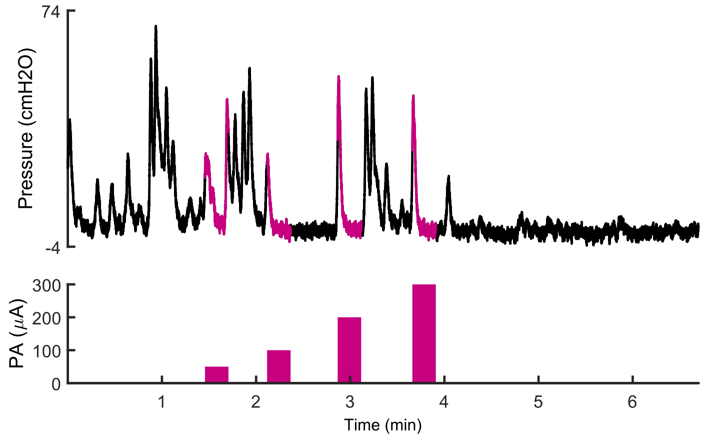

Figure S57. Effect of pulse amplitude of sacral ISMS inhibition (Cat 6, PA0) during complex spontaneous bladder contraction (F = 20 Hz, PW = 100 µs, and stimulation duration = 15 s). Bladder pressure signal (top) and stimulation pulse (bottom).

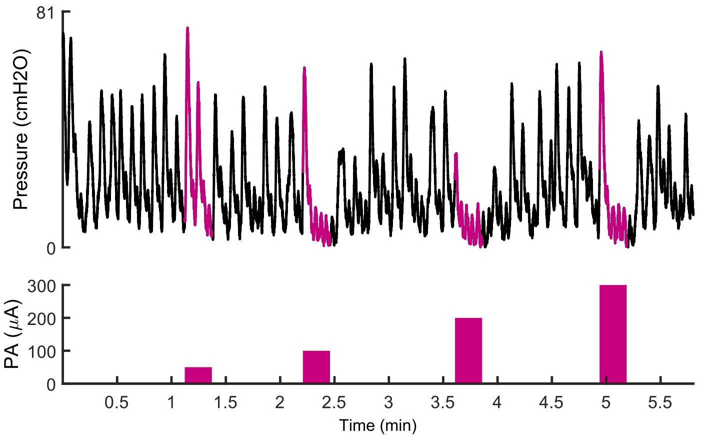

Figure S58. Effect of pulse amplitude of sacral ISMS inhibition (Cat 6, PA2) during complex spontaneous bladder contraction (F = 20 Hz, PW = 100 µs, and stimulation duration = 15 s). Bladder pressure signal (top) and stimulation pulse (bottom).

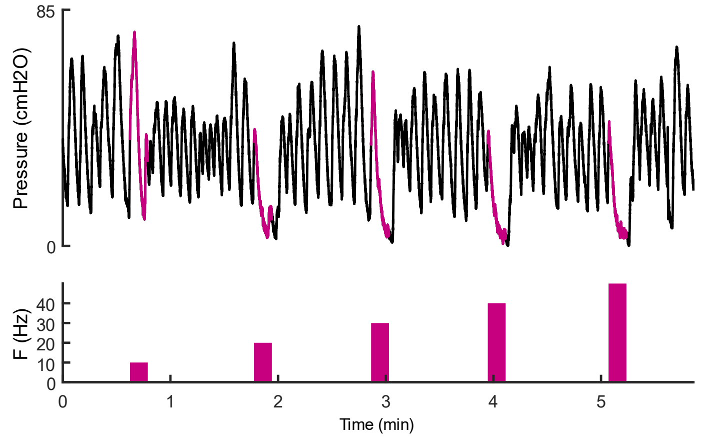

Figure S59. Effect of frequency of sacral ISMS inhibition (Cat 1, F2) during complex spontaneous bladder contraction (PA = 100 µA, PW = 100 µs, and stimulation duration = 15 s). Bladder pressure signal (top) and stimulation pulse (bottom).

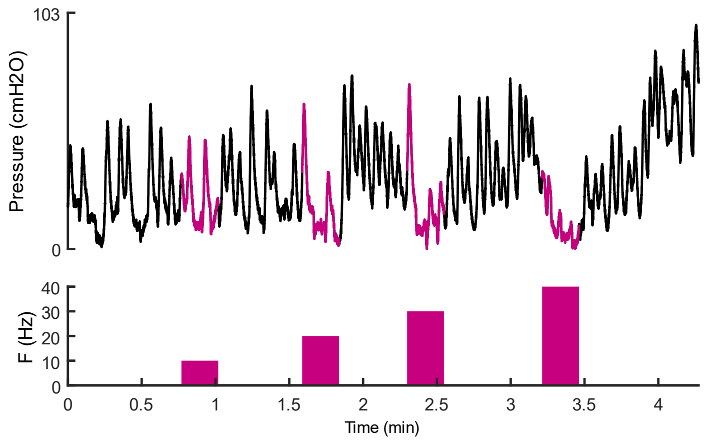

Figure S60. Effect of frequency of sacral ISMS inhibition (Cat 4, F1) during complex spontaneous bladder contraction (PA = 100 µA, PW = 100 µs, and stimulation duration = 15 s). Bladder pressure signal (top) and stimulation pulse (bottom).

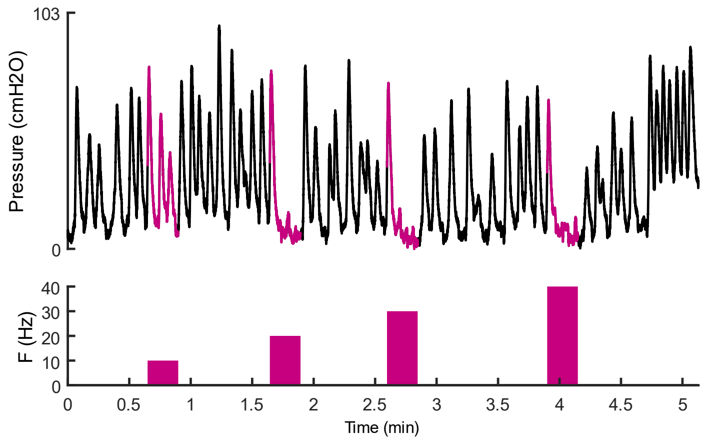

Figure S61. Effect of frequency of sacral ISMS inhibition (Cat 4, F2) during complex spontaneous bladder contraction (PA = 100 µA, PW = 100 µs, and stimulation duration = 15 s). Bladder pressure signal (top) and stimulation pulse (bottom).

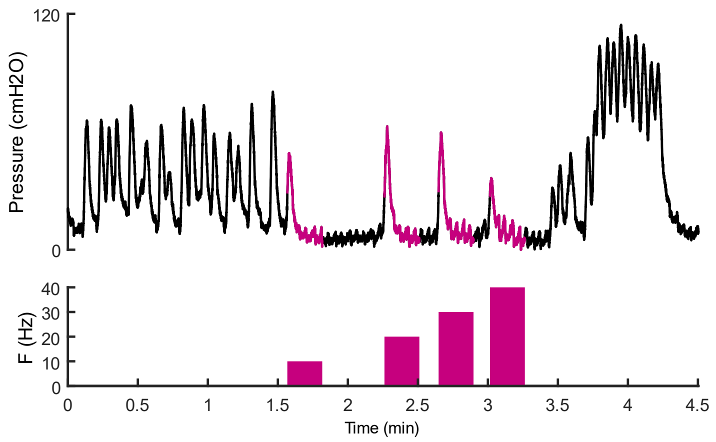

Figure S62. Effect of frequency of sacral ISMS inhibition (Cat 4, F4) during complex spontaneous bladder contraction (PA = 100 µA, PW = 100 µs, and stimulation duration = 15 s). Bladder pressure signal (top) and stimulation pulse (bottom).

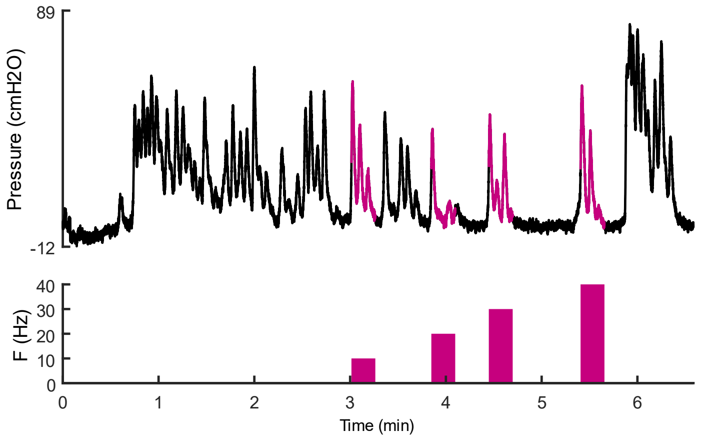

Figure S63. Effect of frequency of sacral ISMS inhibition (Cat 6, F0) during complex spontaneous bladder contraction (PA = 100 µA, PW = 100 µs, and stimulation duration = 15 s). Bladder pressure signal (top) and stimulation pulse (bottom).

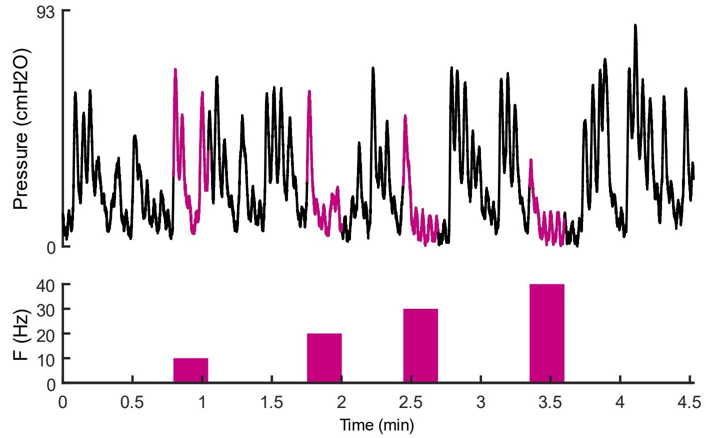

Figure S64. Effect of frequency of sacral ISMS inhibition (Cat 6, F2) during complex spontaneous bladder contraction (PA = 100 µA, PW = 100 µs, and stimulation duration = 15 s). Bladder pressure signal (top) and stimulation pulse (bottom).

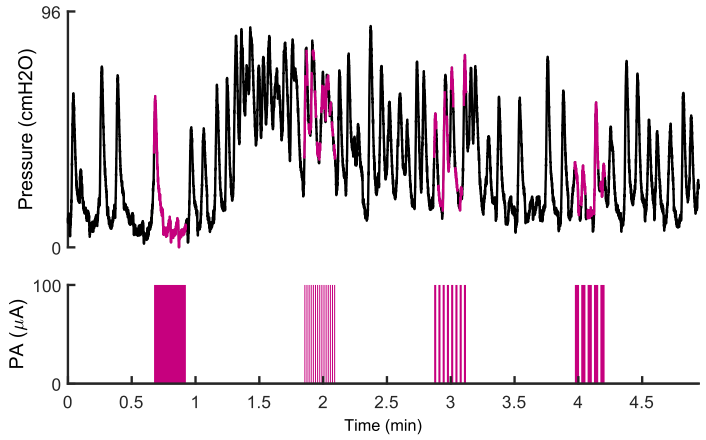

Figure S65. Effect of intermittent pattern of sacral ISMS inhibition (Cat 4, DC1) during complex spontaneous bladder contraction (PA = 100 µA, F = 20 Hz, PW = 100 µs, and stimulation duration = 15 s). Bladder pressure signal (top) and stimulation pulse (bottom).

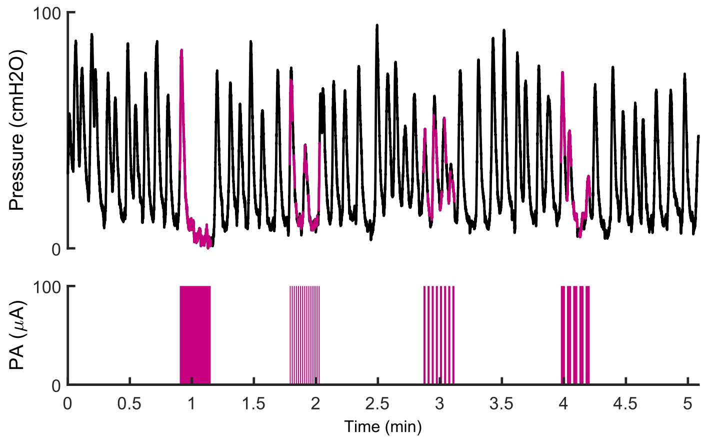

Figure S66. Effect of intermittent pattern of sacral ISMS inhibition (Cat 4, DC2) during complex spontaneous bladder contraction (PA = 100 µA, F = 20 Hz, PW = 100 µs, and stimulation duration = 15 s). Bladder pressure signal (top) and stimulation pulse (bottom).

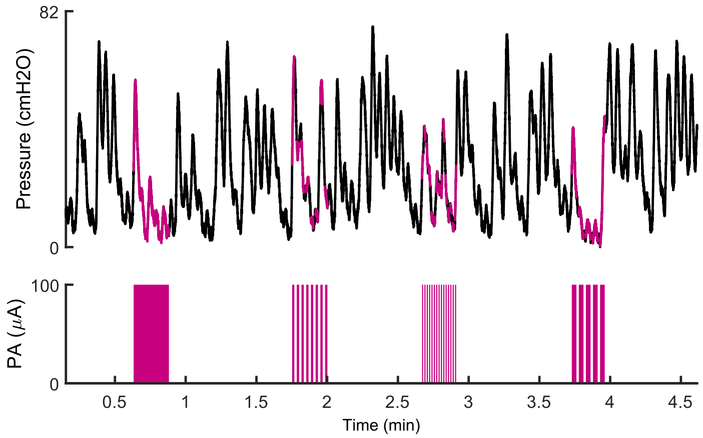

Figure S67. Effect of intermittent pattern of sacral ISMS inhibition (Cat 6, DC2) during complex spontaneous bladder contraction (PA = 100 μA, F = 20 Hz, PW = 100 μs, and stimulation duration = 15 s). Bladder pressure signal (top) and stimulation pulse (bottom).
